# A standardized protocol for quantification of saccadic eye movements: DEMoNS

**DOI:** 10.1371/journal.pone.0200695

**Published:** 2018-07-16

**Authors:** J. A. Nij Bijvank, A. Petzold, L. J. Balk, H. S. Tan, B. M. J. Uitdehaag, M. Theodorou, L. J. van Rijn

**Affiliations:** 1 Department of Ophthalmology, Neuro-ophthalmology Expertise Center, Amsterdam UMC - VUmc, Amsterdam, The Netherlands; 2 Department of Neurology, MS Center and Neuro-ophthalmology Expertise Center, Neuroscience Amsterdam, Amsterdam UMC - VUmc, Amsterdam, The Netherlands; 3 Moorfields Eye Hospital and The National Hospital for Neurology and Neurosurgery, London, United Kingdom; The University of Melbourne, AUSTRALIA

## Abstract

**Objective:**

Quantitative saccadic testing is a non-invasive method of evaluating the neural networks involved in the control of eye movements. The aim of this study is to provide a standardized and reproducible protocol for infrared oculography measurements of eye movements and analysis, which can be applied for various diseases in a multicenter setting.

**Methods:**

Development of a protocol to Demonstrate Eye Movement Networks with Saccades (DEMoNS) using infrared oculography. Automated analysis methods were used to calculate parameters describing the characteristics of the saccadic eye movements. The two measurements of the subjects were compared with descriptive and reproducibility statistics.

**Results:**

Infrared oculography measurements of all subjects were performed using the DEMoNS protocol and various saccadic parameters were calculated automatically from 28 subjects. Saccadic parameters such as: peak velocity, latency and saccade pair ratios showed excellent reproducibility (intra-class correlation coefficients > 0.9). Parameters describing performance of more complex tasks showed moderate to good reproducibility (intra-class correlation coefficients 0.63–0.78).

**Conclusions:**

This study provides a standardized and transparent protocol for measuring and analyzing saccadic eye movements in a multicenter setting. The DEMoNS protocol details outcome measures for treatment trial which are of excellent reproducibility. The DEMoNS protocol can be applied to the study of saccadic eye movements in various neurodegenerative and motor diseases.

## Introduction

The study of eye movements, in particular saccades, is increasingly used as a model for higher-order networks. Besides testing motor control, it can also give insight into neurodegenerative processes and cognitive function [1–4]. These processes remain poorly defined, particularly in the early stage of disease, which makes introduction of new (therapeutical) interventions challenging. This highlights the need for clearly described, objective and validated outcome measures.

The in depth knowledge of the lower order control of eye movements provides unique advantages for quantitative studies compared to other parts of the body. Infrared oculography is a non-invasive and accurate method of recording eye movements [5] and has entered clinical practice in expertise centers. Due to the extensive networks involved in the control of eye movements, both focal and more widespread neuronal processes can be investigated using this infrared oculography [6–8]. There is a large body of literature demonstrating changes of oculomotor performance in diseases such as Alzheimer’s and neurodegenerative dementias, Parkinson’s disease, Multiple Sclerosis (MS), Spinocerebellar ataxia and Huntington’s disease [1–4, 9–13].

Contemporary studies on eye movements generally focus on only one or a few aspects of oculomotor control and protocols differ considerably. Furthermore, there is a lack of transparency on the data analysis, which frequently depends on device specific eye-tracking software. There is shortage on data on validation and reproducibility of outcome measures. This ambiguous reporting limits the ability to critically assess strengths and weaknesses of individual studies. To date there remains a complete lack of multicenter studies. Current research to date highlights the need for a systematic approach and a more generalized protocol of eye movement tests that would be suitable to use by multiple centers.

**Fig 1:**
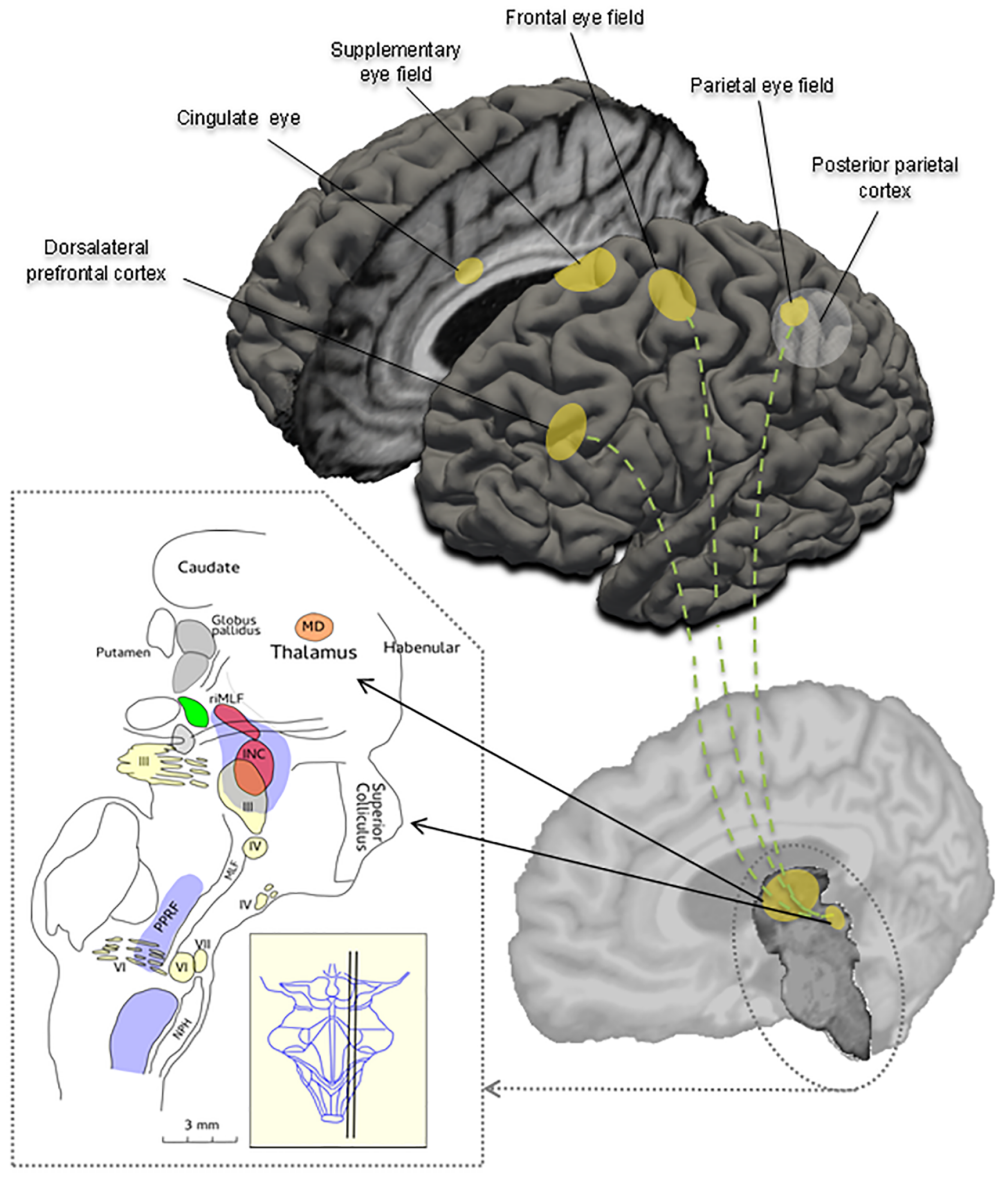
Schematic overview of important cortical regions and brainstem areas involved in the control of eye movements. The sagittal cross section of the brainstem is showing the three oculomotor nuclei (III, IV, VI), the nucleus prepositus hypoglossi (NPH), the interstitial nucleus of Cajal (INC), the superior colliculus, the paramedian pontine reticular formation (PPRF), the medial longitudinal fasciculus (MLF) and the mediadorsal nucleus (MD) in the thalamus. The nucleus raphe interpositus (not shown) lies close to the midline, at the level of the abducens nucleus (VI) [modified from Petzold A, Paine M, Faldon M, Riordan-Eva P, Bronstein AM, Gresty MA, Plant GT. Synchronised paroxysmal ocular tilt reaction and limb dystonia. Neuroophthalmology 2009;33:217–236.].

In this method paper we propose the open-source DEMoNS (Demonstrating Eye Movement Networks with Saccades) protocol, for measuring and analyzing eye movements in a standardized way. The DEMoNS protocol consists of a workable, rapid and easily repeatable sequence of tests, providing insight into the function of different areas in the extensive network of regulation circuits of oculomotor control (Fig 1). The protocol focuses on saccades and fixation because they cover the main oculomotor areas of the brainstem and cerebellum. The dynamic properties of a standard saccadic task have been well delineated in literature [14–16]. Consequently, more complex variations of saccadic tasks can give insight into higher order eye movement control (Fig 1). For several saccadic tasks the neurobiological substrates have been well defined [8, 17]. Prolonged saccadic testing can also be used for demonstration of oculomotor fatigability, which is related to perceived fatigue [18, 19].

For future clinical application and longitudinal follow-up of eye movement measurement, a high level of reproducibility is essential. If tested under the same conditions, saccadic parameters are expected to have little within-subject variation. This is because of the physiological consistency of saccadic dynamics. Consistency is such that it has been proposed that saccadic parameters provide a personal oculomotor signature useful to distinguishes individual subjects [5]. Clinically this enables reliable separation of normal from abnormal saccades [16].

A limitation of these data is the variability in the reported test-retest reliability for saccadic parameters in healthy subjects [5, 20–25]. None of the cited studies included data on the reproducibility of the Versional Dysconjugacy Index (VDI). This is relevant because the VDI is a key variable which may be used to describe an Internuclear Ophthalmoplegia (INO). The VDI represents the ratio of the abducting to adducting eye [26, 27]. There also remains, to date, a lack of reproducibility data of other saccadic parameters, including parameters from a double-step saccadic task and saccadic fatigability task.

In this methods paper, we provide a detailed description of the open-source DEMoNS protocol and descriptive and reproducibility results of healthy subjects, with a view to investigating which parameters are the most robust for various aspects of oculomotor control.

## Materials and methods

We carried out a cross-sectional study across two clinical sites: center one—VU University Medical Center (Amsterdam, The Netherlands, now renamed to Amsterdam UMC—location VUmc) and center two—Moorfields Eye Hospital (London, City Road, United Kingdom). The research followed the tenets of the Declaration of Helsinki. The study was approved by the medical ethical committee of the VU University Medical Center, study number 2015.227. All subjects gave written informed consent.

### Participants

Healthy participants were assessed in the study, with no co-existing ocular or neurological co-morbidity aside from ametropia, in these subjects measurement was performed with correction (glasses or contact lenses). At center one subjects were assessed on two occasions (more than one day apart). In order to explore the external validity, we additionally assessed healthy subjects at center two.

### Set-up

The protocol and equipment used was standardized across both centers. Binocular oculography was performed using an Eyelink (SR Research Ltd., Mississauga, Canada) eyetracker and at maximum sampling frequency. The Eyelink 1000 Plus with data sampled at a frequency of 1000 Hz was used in in center one and the Eyelink 1000 with data sampled at a frequency of 500 Hz was used in center two. Both devices detect the pupil and corneal reflection with infrared light.

**Fig 2:**
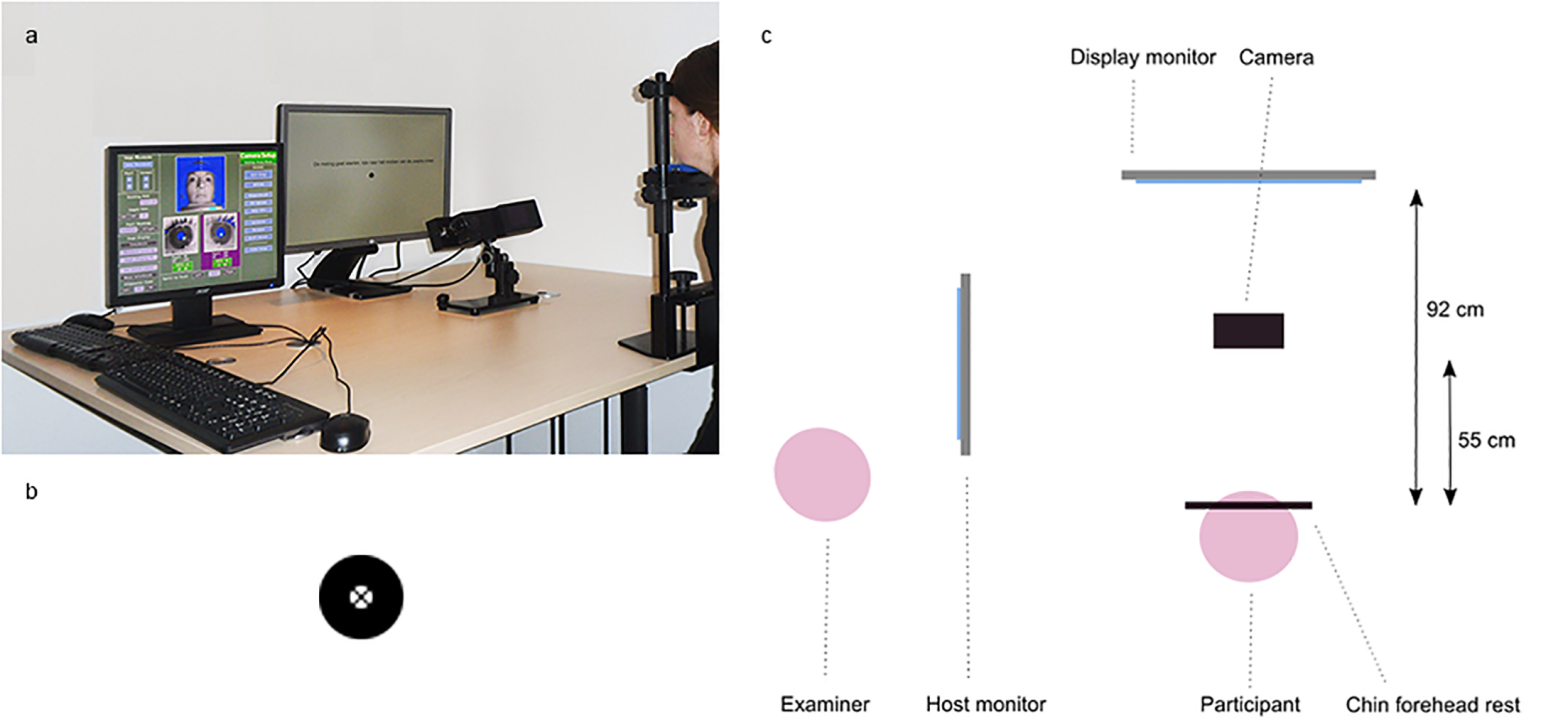
DEMoNS set-up. a. Picture of the device set-up. Participants were seated in front of a display monitor, with their head stabilized with a chin and forehead rest. The camera was located in front of the chin rest, just below the line of sight. b. Target used in the measurements, a black circle with a black cross in the center of the circle. c. Schematic overview of the set-up (view from above). The participant in the chin and forehead rest is located 92 cm in front of the display monitor and 55 cm in front of the camera. The examiner is located on the left of the participant and can check stability of the signal and performance of the participant on the host monitor.

In center one, participants were seated at 92 cm (eye-monitor distance) in front of a display monitor (HP Elite Display E241i, 24 inch, resolution of 1024x768 pixels used). The head was stabilised by means of a chin and a forehead rest. The desktop mount (with the camera) was located 50–55 cm in front of the chin rest, just below the display monitor (Fig 2). All experiments were performed under dim lighting conditions (20 to 50 Lux). In center two, the set-up was similar, with a display monitor (Iiama ProLite E2207WS, 22 inch) and an eye-monitor distance of 78 cm, creating the same visual angles during the tasks.

Built-in algorithms provided by the manufacturer of the eye tracker were used for calibration and validation procedures, with static targets at known horizontal and vertical eccentricities (maximum of 15 degrees). A 9-point calibration procedure was used, with quality control by the operator for each calibration target. The accepted mean error for fixation variation was less than 1.0 degrees with a maximum error of the series less than 1.5 degree of visual angle. The calibration was reliable in each subject without any need to repeat the procedure.

The target used for calibration, validation and the assessments, consisted of an outer black circle, an inner white circle (respectively 0.75 and 0.20 degrees of visual angle), with a black cross in the center of the circle presented on a white background (Fig 2). This was with the intent of creating a high contrast target that would also be clearly visible in subjects with visual impairments. Subjects were instructed to fixate the midpoint of the circle (which corresponded to the midpoint of the cross).

To ensure each participant received the same information and understood the task sufficiently, standardized instructions were given to the participants and example and practice trials performed before the tasks, as indicated in S1 File. During and after these trials feedback was given, which comprised repeating the instructions. Between the trials there were short resting periods (approximately five seconds). The total duration of the experiment (excluding the instructions) was 21 minutes.

### Measurement protocol

A proposed sequence of assessments was developed to test different areas in brainstem, cerebellum and brain that are part of the complex network involved in eye movements (Fig 1).

**Fig 3:**
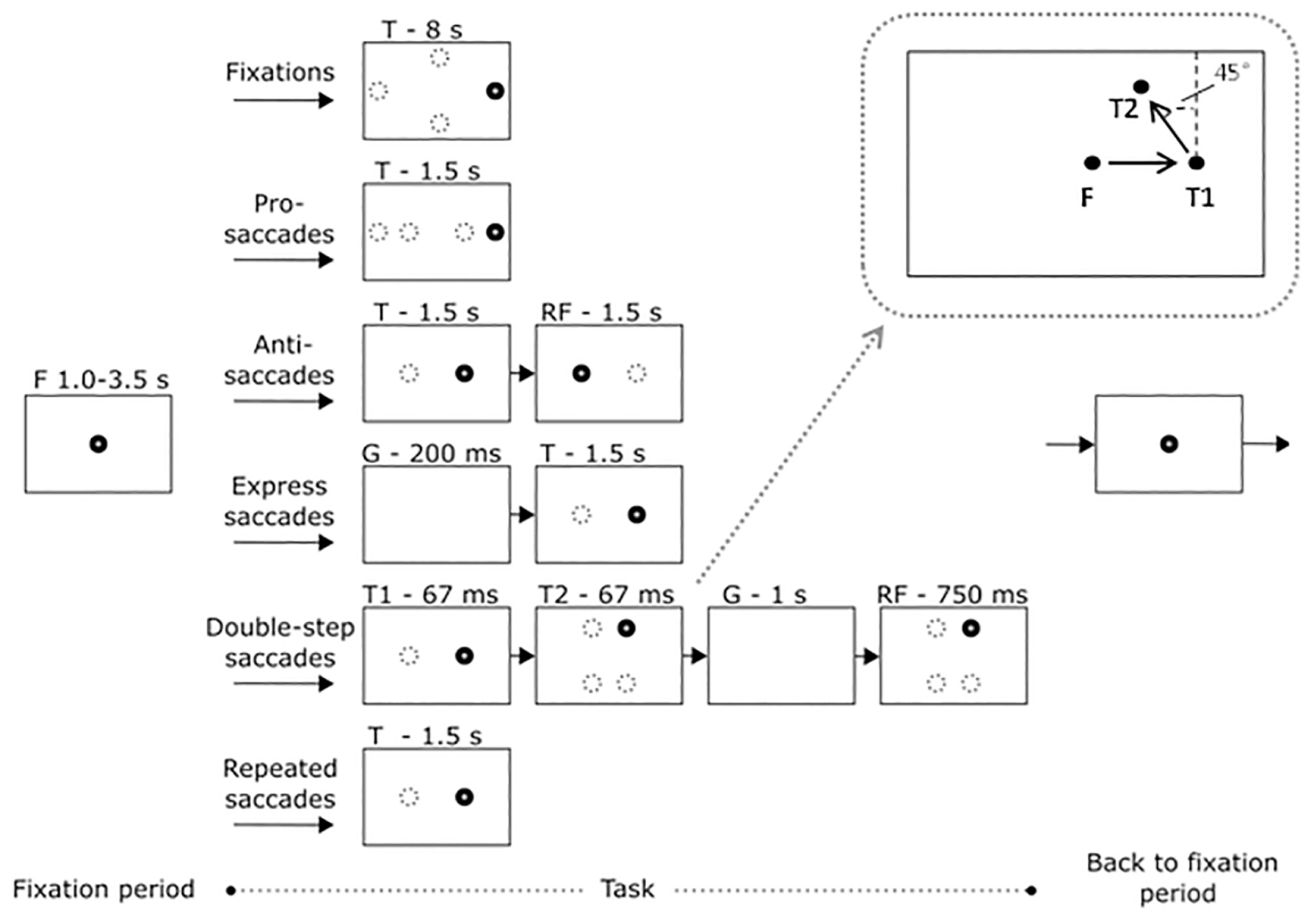
Assessments in the DEMoNS protocol. All assessments start with a fixation period (F) at a central target (black dot), with a random duration between 1.0 and 3.5 seconds. After this, depending on the task, a gap period (G), one or two targets (T, T1, T2) and refixation targets (RF) are appearing (black dots) of different durations. The circles with the dashed lines show the other possible locations of the targets in this task. Afterwards, the central target re-appears, and the task will restart. All target steps (fixation position to target and target one to target two) encompass 8 degrees of visual angle, except for the vertical target steps in the fixation task (10 degrees of visual angle) and the most eccentric target steps in the pro-saccadic task (15 degrees of visual angle). In the top right corner, an enlarged example of one possible combination of target locations of the double-step saccadic task is shown.

The protocol included six domains: fixation, pro-saccades, anti-saccades, express saccades (gap paradigm), double-step saccades and repeated pro-saccades. The tasks are summarized in Fig 3 and explained in more detail in S1 File.

### Data-analysis

**Fig 4:**
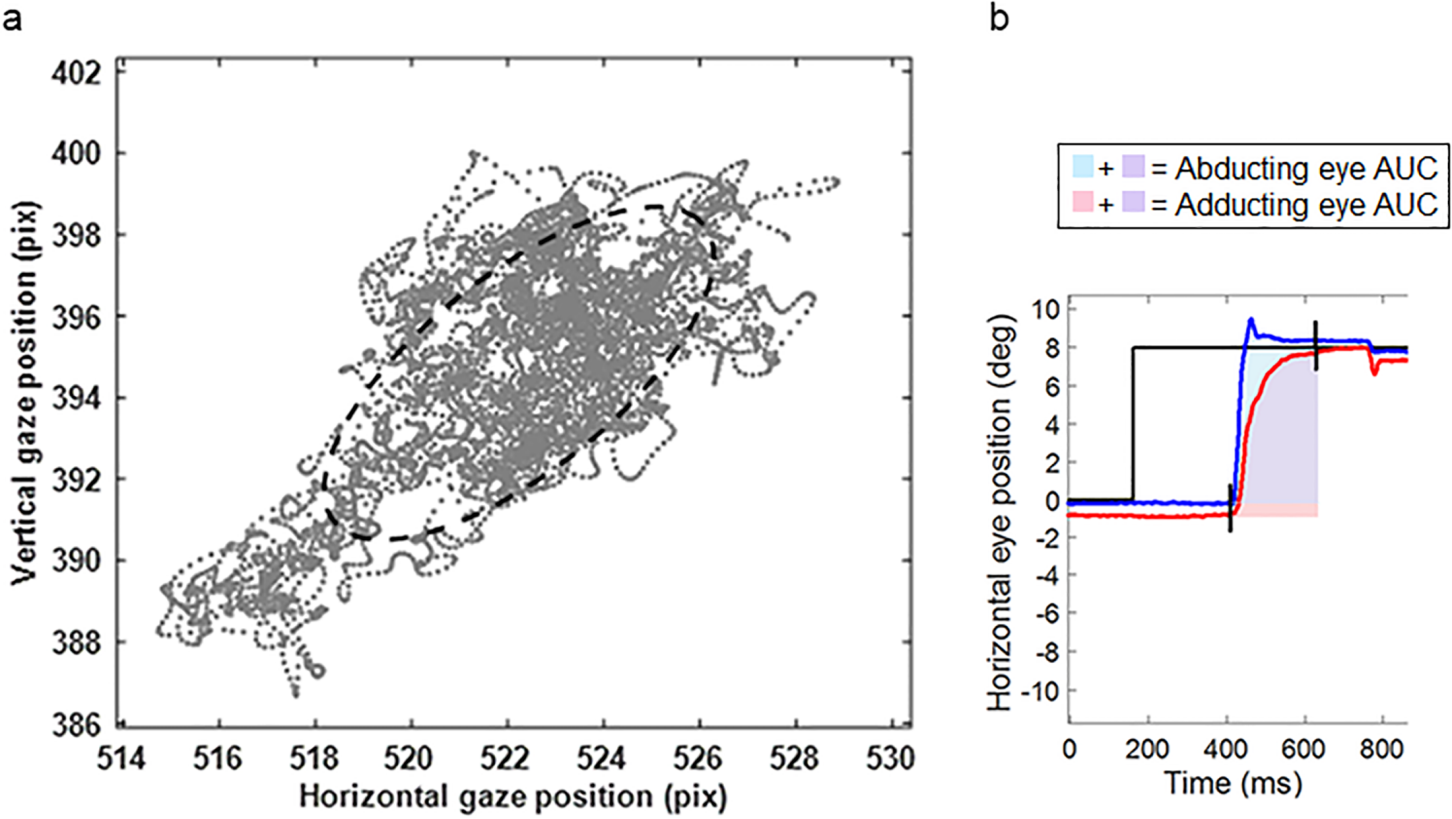
Example of two parameters of the DEMoNS protocol. a. Scatterplot of left eye gaze positions of one subject (JNB) of the first central fixation trial. The 68% Bivariate Contour Ellipse Area (BCEA) is represented by the dashed line, which is the area of a bivariate ellipse encompassing 68% of the highest density samples. The arrows are indicating the distance of 0.1 degree of visual angle in the horizontal and vertical dimension. b. Schematic representation of the area under the curve (AUC) of the horizontal saccadic trajectory of a rightward saccade. The blue line represents the right eye and the red line represents the left eye. The AUC is assessed from the first starting saccade (left or right eye) until the last ending saccade (left or right eye). In this period the area is calculated for both eyes separately by summing the horizontal eye position at every time point minus the horizontal start position of the saccade.

The default sample filter of the Eyelink system is a heuristic low-pass filter [28, 29]. For semi-automated off-line analysis of the eye movement data, an in-house program written in Matlab (Mathworks, inc., Natick, MA) was used. For development of the analysis steps, visual inspection of the data was performed to optimize and check different settings, noise filters and thresholds. This allowed the developed protocol to automatically and correctly supress noise and detect events in the data of both centers. Criteria for quality control of saccades and other events were implemented for every task. A brief summary of the data analysis is provided in Table 1, with a detailed description in S2 File. An example of one parameter of the fixation task is shown in Fig 4a, the Bivariate Contour Ellipse Area (BCEA) and one parameter of the pro-saccadic task in Fig 4b, the Area Under the Curve (AUC) of the horizontal saccadic trajectory. Where there was automated removal of more than 50% of the saccades (or fixation samples in the fixation task) in a specific task, the data for that particular task was excluded from further analysis.

**Table 1:** Summary of analysis steps of the eye movement data.

Analysis step		Description
**Pre-processing**	1. Filtering	Maximally flat 2^nd^ order low-pass filter without delay and 9-sample wide edge enhancing smoothing kernel
	2. Artefact removal	Removal of samples between 0.10 seconds before and 0.25 seconds after a blink artefact
**Saccadic detection**	1. Detect approximate saccadic intervals	Samples with acceleration >2.576*SD of acceleration distribution
	2. Merging of saccadic intervals	Intervals closer than 20 ms
	4. Detect onset and offset of saccade	From highest velocity check consecutive samples in backward and forward direction, onset or offset if 1 of 3 criteria satisfied: 1. Sample to sample direction deviation from main direction (which is the sample-to-sample direction at highest velocity) of >60 degrees 2. Sample to sample directional change between two adjacent samples of >20 degrees per ms of sample duration 3. Velocity of <5 degrees/second
	5. Minimal saccadic amplitude and duration	Amplitude of >0.15 degrees and duration of >8 ms
	6. Determine the main saccade	Saccade with the highest peak velocity of a saccadic interval
**Parameters calculation**	Fixation task	Fixation periods: mean and SD of gaze, vergence and velocity. Median and IQR of total velocity. Bivariate contour ellipse area. Linear fit coefficient and standard error of estimate. Number of saccadic intrusions (divided in square wave jerks (SWJ) and saccades) per second. Mean amplitude of saccadic intrusion. Mean inter-SWJ interval.
	Pro-saccadic, express saccadic and repeated pro-saccadic tasks	Centrifugal saccades: peak velocity, peak acceleration, gain, latency, firstpass gain (FPG), area under the curve of the saccadic trajectory (AUC). Versional dysconjugacy index (VDI) of: peak velocity, peak acceleration, FPG and AUC.
	Anti-saccadic task	Centrifugal saccades: peak velocity, peak acceleration, latency, gain, X error, proportion of errors. Final eye position: gain, X error.
	Double-step saccadic task	First and second saccade: peak velocity, peak acceleration, latency/intersaccadic interval, amplitude/gain. Final eye position: gain, X error, Y error. Proportion of correct, acceptable, contraversive shifted and late double-step saccades. Proportion of first saccade to second target

### Statistics

Analysis was performed using SPSS version 22.0 (SPSS, Inc., Chicago IL). Mean, standard deviation (SD) and range of the two measurements were calculated for the different parameters. To compare differences between saccades within the two measurements (within one task or between different tasks), the paired sample t-test was used, after checking the normality of the distribution visually. A probability level of alpha<0.05 was considered as significant. To quantify the test-retest scores, the intra-class correlation coefficient (ICC) was calculated. The ICC was based on a two-way mixed ANOVA model for single measures, testing absolute agreement between the two measurements. The test reproducibility is defined as excellent for an ICC greater than 0.90, good for 0.75–0.90, moderate for 0.50–0.75 and poor if <0.50 [30]. A high ICC indicates a high inter- versus intrasubject variation. Furthermore, the coefficient of variation (CV) and the coefficient of repeatability (CR) were calculated. The CV and CR were both derived from the within-subjects standard deviation (s_w_), also called the standard error of measurement. The s_w_ was calculated by taking the square root of the mean variance of the two measurements of every subject. Next, the CV was calculated by expressing the S_w_ as a percentage of the mean of the two measurement [31, 32] and the CR by multiplying the S_w_ by 2.77 (= √2 * 1.96) [33–35]. Consequently, the CR quantifies the degree of absolute agreement in the same units as the measurement outcome.

## Results

### Participants

In center one, 19 subject were included, with a mean age of 40 (range 23–69) of which 53% of female gender. In center 2, 12 subjects were included, with a mean age of 34 (range 24–53) years old, of which 58% of female gender. Three subjects were measured in both centers. In all subjects complete measurement and analysis were accomplished.

### Center one—Infrared oculography

In S1–S6 Tables, the calculated parameters of every task are listed, with the mean, SD and range of the first and second measurement and the ICC, CR and CV.

#### Fixation task

None of the fixation periods of the measurements failed quality control, therefore 10 fixation periods per measurement were analysed. On average, 2206 of 70.000 (3.2%) fixation samples per measurement had to be discarded. As expected for healthy subjects, the majority of the fixation stability and saccadic intrusion parameters showed very little variation between the subjects, with SDs and BCEA of gaze position and vergence of less than half a degree of visual angle in all subjects. The median of the total eye velocity during fixation was 3.0 deg/s, and showed high reproducibility between the two measurements (ICC 0.89). Macro Square Wave Jerks (MSWJs) weren’t present in any of the subjects, but a few large intrusive saccades (mean of 0.01 per second) were documented. Furthermore, Square Wave Jerks (SWJs, <4 degrees of visual angle) and microsaccades were found with a mean of 0.20 and 0.62 per second respectively.

#### Pro-saccadic task

**Fig 5:**
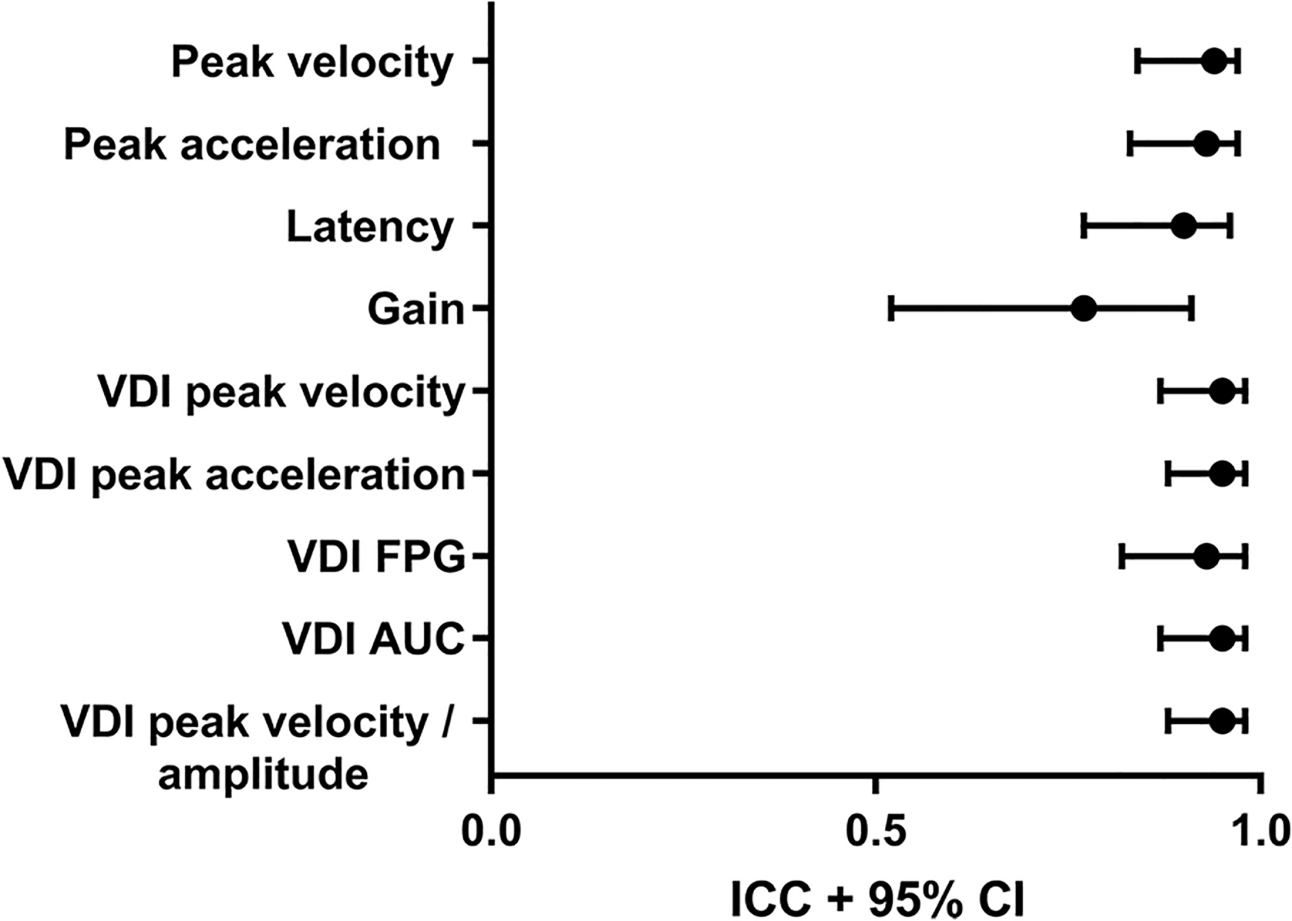
Intra-class correlation coefficients of pro-saccadic task. Intra-class correlation coefficient (black dots) with 95% confidence interval (error bars) of parameters of the pro-saccadic task.

On average 2.7 of 60 (4.4%) centrifugal saccades per measurement failed quality control, mainly due to blinking. Excellent ICCs were found for peak velocity, latency and most VDI parameters. Good ICCs were found for gain and other VDIs, as the rightward VDI of the peak acceleration. The ICCs with a 95% confidence interval are shown for pro-saccadic parameters in Fig 5.

**Fig 6:**
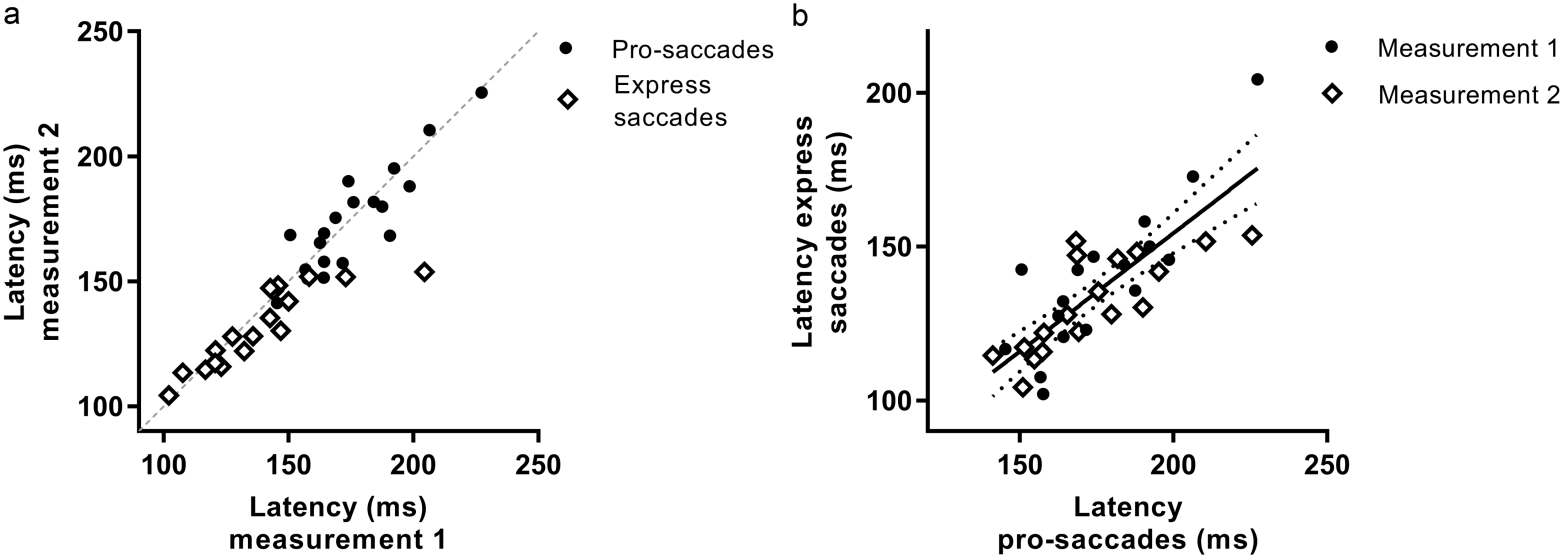
Latency of pro-saccades and express saccades. a. Latency of pro-saccades (black dots) and express saccades (white diamonds) of second measurement against first measurement of all subjects. The dotted line corresponds to absolute agreement between the two measurement. b. Latency of first measurement (black dots) and second measurement (white diamonds) of pro-saccades against express saccades. The linear fit (black line) with 95% confidence interval (dotted lines) of all measurements is shown.

The between subject variability for saccadic parameters as peak velocity and latency was considerable (SD of 45 deg/s and 22 ms respectively). In contrast, all saccade pair ratios (VDIs) of the participants showed a narrow distribution (SD for all VDIs 0.11 or below). As expected, based on the main sequence relationship of saccades [14], velocity and acceleration for 15 degrees saccades were higher than for 8 degrees saccades (mean difference 57 ±16 deg/s and 3203 ±1537 deg/s/s respectively, p<0.001). Also, latency was longer (p<0.001) and gain lower (p<0.001) when comparing 15 with 8 degrees saccades. In Fig 6a, the latency of the first and second measurement of 8 degree saccades of all subjects is shown. For VDI peak velocity there was no significant difference between 15 and 8 degrees saccades. In contrast, the VDI peak acceleration was higher (mean difference 0.01 ±0.03, p = 0.005) and VDI first-pass gain (FPG) and VDI area under the curve (AUC) values were lower when comparing 15 with 8 degrees saccades (mean difference -0.03 ±0.03 and -0.02 ±0.03 respectively, p<0.001). When calculating the VDI FPG, on average 13.5 (23.6%) saccades per measurement could not be taken into account, because this ratio could not be determined in hypometric saccades (undershooting the target).

#### Anti-saccadic task

In the anti-saccadic task, on average 0.8 of 40 (2.1%) centrifugal saccades per measurements failed quality control. Peak velocity, peak acceleration, latency, proportion of errors and horizontal error of the end position of the anti-saccades showed good to excellent reproducibility. For the gain of the anti-saccades and latency of incorrect pro-saccades moderate ICCs were found.

**Fig 7:**
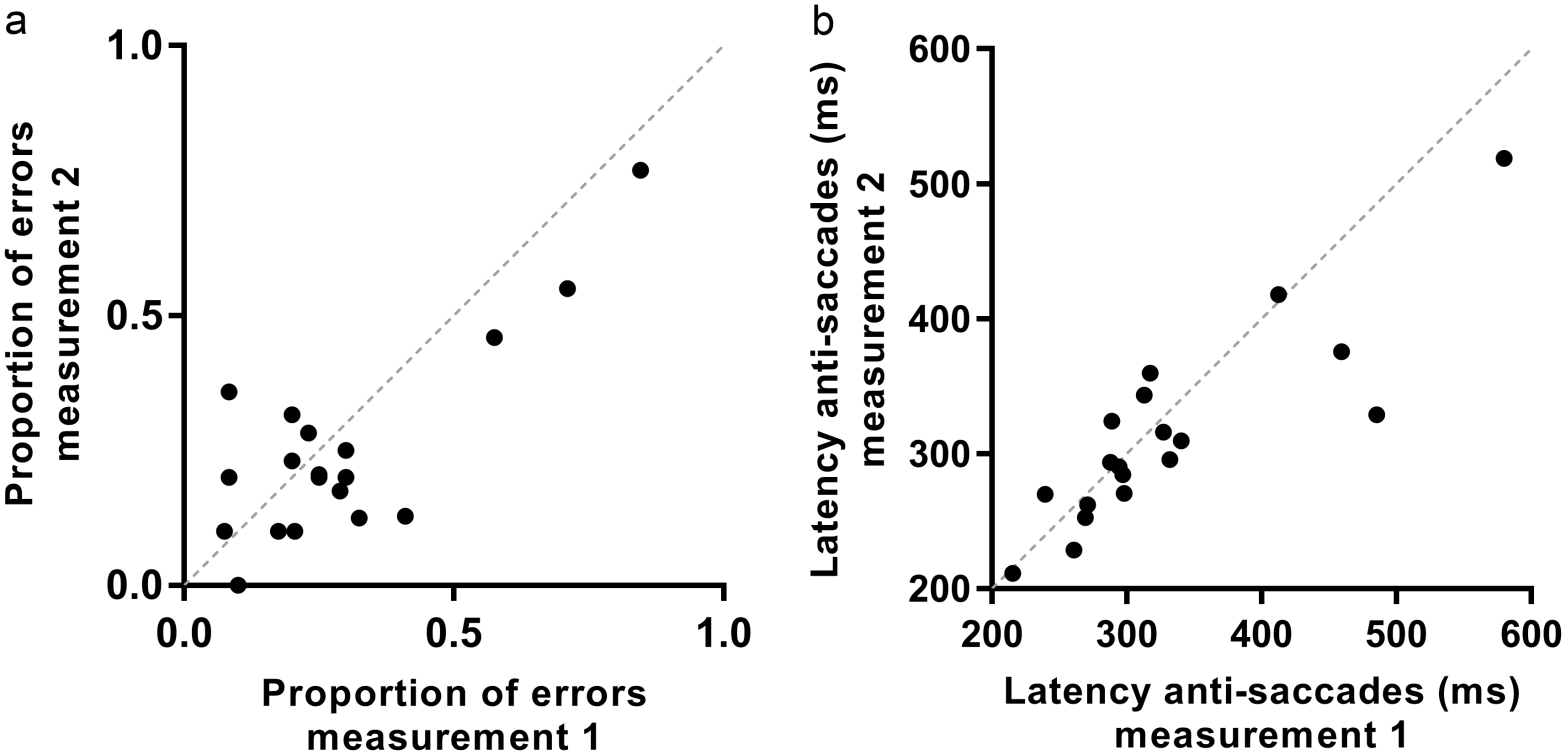
Proportion of errors and latency of anti-saccades. a. Proportion of errors of second measurement against first measurement of all subjects (black dots). The dashed line corresponds to absolute agreement between the two measurement. b. Latency of correct anti-saccades of second measurement against first measurement of all subjects (black dots). The dashed line corresponds to absolute agreement between the two measurement.

The proportion of errors for every subject of first against second measurement is shown in Fig 7a. On average over the two measurements, the proportion of errors was 0.27, with a considerable variability between subjects (total range of two measurements 0.00–0.85). Furthermore, subjects made significantly more errors to right-sided targets than to left-sided targets (mean difference 0.10 ±0.13, p<0.001). Latency of correct anti-saccades (Fig 7b) was significantly longer than latency of incorrect pro-saccades (mean difference over 110 ±79 ms, p<0.001).

#### Express saccadic task

For the express saccadic task, 18 subjects passed quality control and for these subjects on average 2.7 of 30 (8.9%) centrifugal saccades per measurement failed quality control.

Peak velocity, peak acceleration, latency and gain showed good reproducibility. The latency of the second against the first measurement is shown in Fig 6a. In Fig 6b, the relationship between latency in the pro-saccadic and express saccadic task is shown. In both the first and second measurement, the subjects made saccades with significant shorter latencies compared with the 8 degrees saccades of the pro-saccadic task, with a mean difference of 40 ms (±13 ms, p>0.001). The gap paradigm used in this task is expected to elicit shorter latency saccades, the above results comprised all saccades included by quality control. One subject (AP) was excluded from this task because of anticipatory responses, resulting in removal of >50% of the saccades.

#### Double-step saccadic task

**Fig 8:**
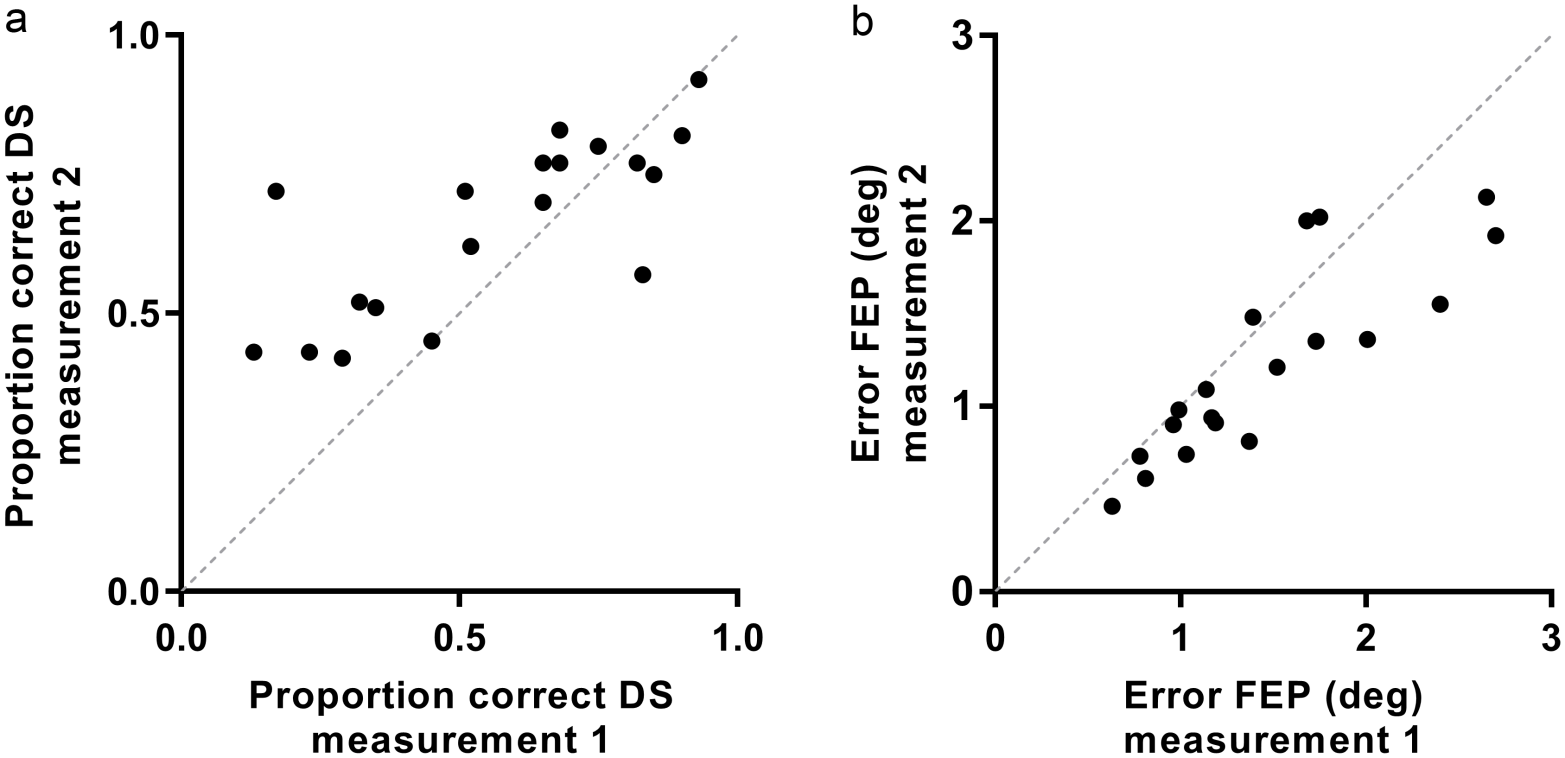
Proportion of correct double-step saccades and error of the final eye position. a. Proportion of correct double-step saccades (DS) of second measurement against first measurement of all subjects (black dots). The dashed line corresponds to absolute agreement between the two measurement. b. Error of the final eye position (FEP) in degrees of visual angle of double-step saccades of second measurement against first measurement of all subjects (black dots). The dashed line corresponds to absolute agreement between the two measurement.

In the double-step saccadic task, on average 0.3 of 60 (0.5%) double-step saccades were discarded based on blinking or signal disturbances and another mean of 12.3 (20.6%) per measurement did not fulfil quality control criteria for double-step saccades. This second group of saccades was not taken into account for calculating parameters, but was included for the denominator when calculating the proportions of (correct or other) double-step saccades. The error of the final eye position after double-step saccades was below 3 degrees of visual angle for all subjects (Fig 8b), with good reproducibility (ICC 0.77) between the measurements. On average, the majority of double-step saccades was correctly performed (61 ±22%), with moderate reproducibility (ICC 0.63). There was, however, a large variability between subjects (Fig 8a). On average, performance improved in the second measurement, although the difference in correct double-step saccades between the two measurements was not statistically significant. Correct performance of double-step saccades was not significantly different between left- and right-sided targets (mean difference 2.3 ±21%, p = 0.50).

#### Repeated pro-saccadic task

In the repeated pro-saccadic task, on average 1.2 of 30 (4.1%) centrifugal saccades per measurement failed quality control. Latency, peak velocity, peak acceleration and most VDIs showed good to excellent reproducibility and gain moderate reproducibility.

In both measurement, the saccades were more hypometric and slower compared with the 8 degrees saccades of the pro-saccadic task, with a mean gain difference of 0.03 (±0.03, p = 0.001) and mean peak velocity difference of 20.8 deg/s (±17.9, p<0.001). The latency was not significantly different from the latency in the pro-saccadic task.

### Center two—Infrared oculography

**Fig 9:**
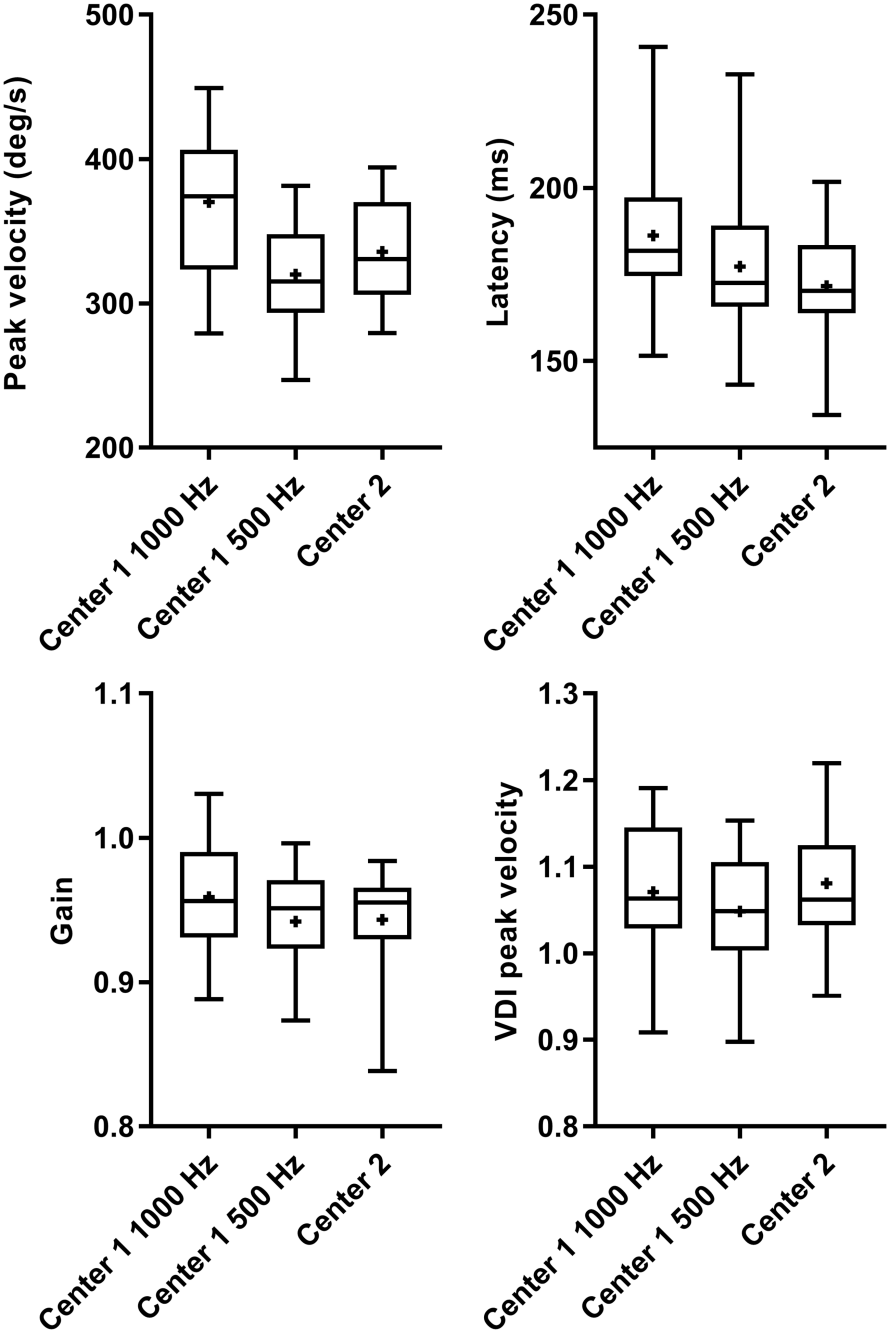
Comparison of pro-saccadic parameters of center one and center two. Box (25–75%)-and-whisker (0–100%) plots from pro-saccadic parameters of center one (both at sampling a frequency of 1000 Hz and downsampled to 500 Hz) and center two. The horizontal line in the box is plotted at the median and the plus sign indicates the mean.

**Fig 10:**
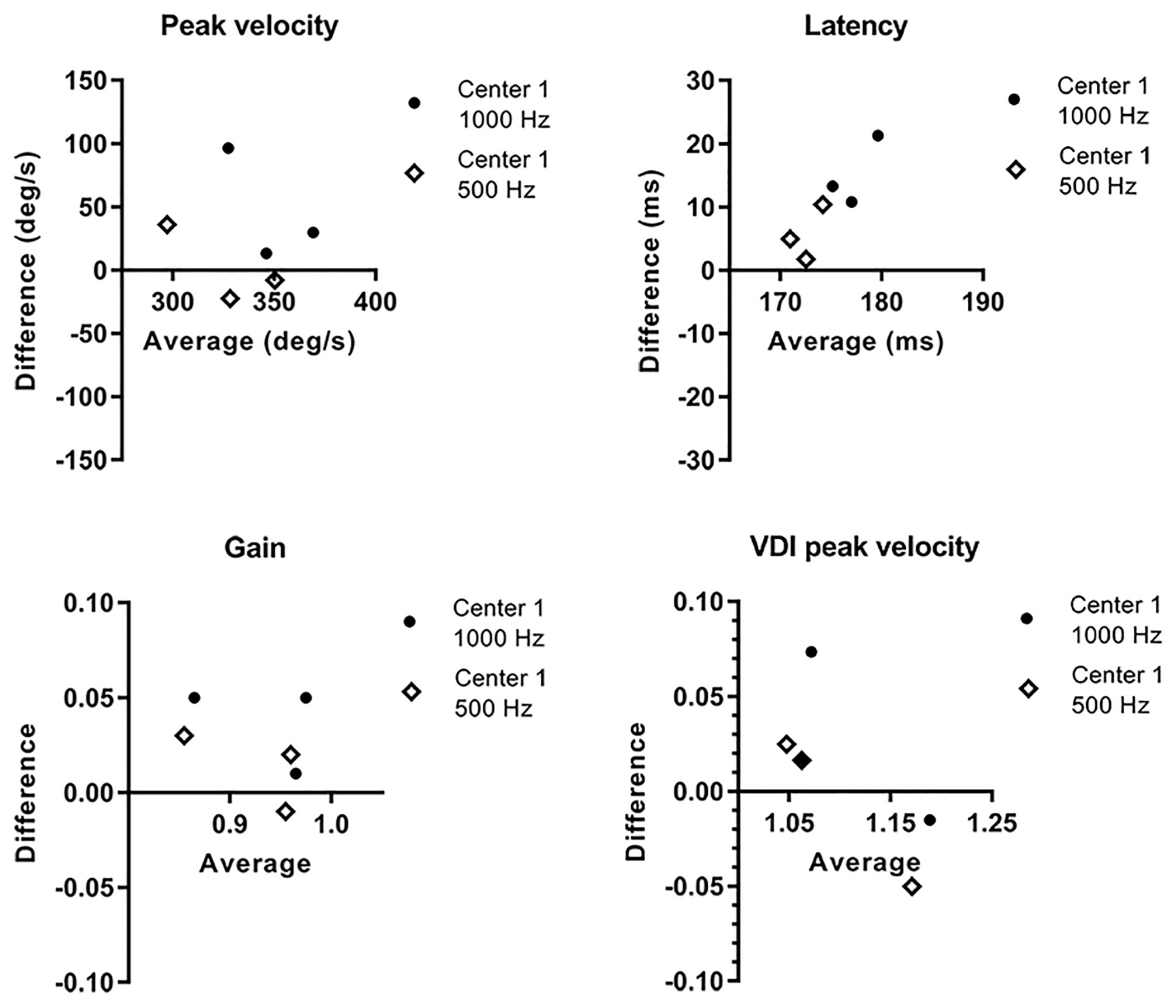
Bland Altman plots of pro-saccadic parameters of centerone and two. On the x-axis, the mean of the two measurements for the three subjects tested at both centers is shown. On the y-axis the difference (center one minus center two) of the two measurements is shown. Both comparison with the original sampling frequency of center one of 1000 Hz (black dots) and downsampled to 500 Hz (white diamonds) is shown.

The implementation of the measurement protocol in center two was straightforward. Overall, the range of the described parameters was similar to center one (S7 Table)–the parameters even more closely resembled center one when the data from center one was downsampled to 500 Hz. As an example the results of a few pro-saccadic parameters of center one (both at 1000 and 500 Hz) and two are shown in Fig 9. Bland Altman plots for the same parameters of the three subjects who were measured in both centers are shown in Fig 10. In center two, the data export was corrupted for one subject and for another subject (AP) the express saccadic task was excluded, due to anticipatory responses (similar to measurement of this subject in center one).

## Discussion

This study provides a standardized, transparent, rapid (<25 minutes) and open-source method for measuring and analyzing a range of relevant saccadic and fixational eye movements. With the described quality control settings, the algorithms were able to automatically and reliably calculate the parameters of interest. The basic saccadic parameters as peak velocity, latency and VDIs, especially of the pro-saccadic task, showed good to excellent reproducibility. Saccadic characteristics followed main sequence relationships and gaze stability in the fixation task were similar to values reported in literature [8, 14, 36, 37].

As expected in some of the tasks, e.g. the fixation task, the between subject variability was very low, because all measured subjects were capable of holding steady fixation. The ICC is lower in homogeneous population as it expresses within-subject variability in proportion to total variability. Consequently it is difficult to directly compare ICC values between tasks or parameters [38]. The CR, which is also referred to as the Smallest Real Difference (SRD), lends itself for easier (practical) interpretation in these tasks, as the absolute difference between any two future measurements is expected to be at most the value of the CR (in 95% of the cases). Higher differences can be indicative of real differences between healthy subjects and patients or a real change over time in longitudinal or treatment studies.

Reproducibility values decreased with increasing complexity of a task and especially when expressing performance of a task (e.g. number of errors in anti-saccadic task, proportion of correct double-step saccades). The within subject variability was low enough to differentiate between performances of the subjects. This, however, raises the question of whether these parameters are suitable for testing in patients on an individual level. In this study, the mean proportion of errors in the anti-saccadic task by normal subjects was higher than previously reported, although the range between participants and between studies is very high [39, 40]. Variations could be related to the protocols used or practice, a possible explanation in our protocol could be the additional task of re-fixation of the target after the anti-saccade, which makes the task more complex. On the other hand, these findings could just as well fall within normal subject to subject differences, given the high variability in neuropsychological performance in healthy adults [41]. Further studies with large groups of patients and matched healthy controls are required to elucidate the range of normal versus pathologic scores. This could also aid in exploring the cause for the unexpected directional asymmetry in anti-saccadic error, which could be a coincidence, related to set-up or have a physiological background [42].

An international expert meeting in 2013 concluded by suggesting a research protocol for testing of saccades and anti-saccades [43]. In line with the recommendation made in 2013 the DEMoNS protocol incorporated the core stimulus parameters, trial parameters and outcome measures. Extending on this work the DEMoNS protocol focused on brevity, reliability and reproducibility in order to become more suitable for implementation in a multicenter clinical setting. As a result our DEMoNS protocol included fewer number of trials for pro- and anti-saccades. In order to test oculomotor fatigability the DEMoNS protocol included a repeated pro-saccadic task rather than the previously described prolonged pro-saccadic task [18, 19]. Our study showed that reproducible results can be achieved with fewer modifications which saves time and makes the protocol easier to implement in clinical practice. In addition, a fixation, express saccadic and double-step saccadic task were incorporated to test more aspects of oculomotor control. The double-step saccadic task is expected to test corollary discharge (an internal copy of an impending motor command) and by this higher order oculomotor control by different regions [44–49].

Eye movement measurements with infrared oculography are increasingly used in different domains of research, mostly investigating saccadic characteristics. The accuracy and validity of these outcomes, however, dependent on the choice of algorithms used for parsing the data and saccadic detection. The majority of previous studies relied on algorithms integrated in the eye-tracking software and did not explicitly evaluate this decision. Custom made algorithms are not always publicly accessible for researchers, which adversely affects data transparency. Furthermore, there is not yet much agreement what combination of equipment and settings is the best. This makes it difficult to generalize results from one research institute to another and to compare results between studies. In the elegant recent review of Andersson et al. [50], an attempt was made to compare different algorithms for saccadic detection. Ten different algorithms were evaluated, and the results were compared with assessments of two human raters. The authors concluded that it was not possible to identify one perfect algorithm and instead highlighted the strengths of the ‘LNS algorithm’ [51] for detecting saccades and post-saccadic oscillations. The inter-rater degree of agreement was considered good (Cohen’s Kappa’s for saccades between the algorithm and the raters ranging from 0.75–0.81 for different stimuli). Consequently we based the saccadic detection in our analysis on the LNS algorithm. We would be hesitant to extrapolate these data from healthy young adults to patients. For future studies in pathology it will be important to scrutinize data manually as well.

Another issue to be taken into account is the difference in measurement devices between centers. When comparing the data between the two centers in our study, the Welch spectrum of the raw data (S1 Fig) suggests a relevant difference in internal data processing and filtering of the two devices, even though these devices are from the same manufacturer. Furthermore, comparison of gaze, velocity and acceleration signals at different sampling frequencies (S2 Fig) supports our finding that measuring at different sampling frequencies results in differences in relevant parameters. Likely, due to better signal power in high temporal frequencies which are missed when sampling at a low frequencies. These limitations need to be taken into account when directly comparing data from different devices as well as equalizing sampling frequencies (which can be done directly at the time in the measurement or by downsampling afterwards). Another possible contributing factor and important issue in all modern eye-tracking devices is temporal aliasing, which can occur when the digital camera of the device samples the moving images to a discrete signal. However, the commonest scenario is that there is no information provided by manufacturers on anti-aliasing procedures of the original non-discrete signal. Altogether, when describing eye movements in diseases, a matched healthy control group measured with the same device is advisable.

When choosing parameters to describe, we recommend taking into account the reproducibility values given in this paper. This should guide the set-up of future studies aiming to investigate the magnitude of differences between patients and healthy controls. This protocol will be assessed as a solid and sensitive assessment for motor (dys)function, cognitive control and fatigue in MS patients. Our preliminary data has already shown some consistent results in this patient group [52, 53]. Extrapolating from this preliminary data, the DEMoNS protocol is useful in the study of a demyelinating disease.

We have shown that our standardized protocol can be used as a repeatable and reliable method for measuring a wide range of oculomotor parameters. This open-source protocol will be useful in a multicenter clinical and research setting, as it is applicable and relevant to a range of diseases [1–4, 9–11].

## Supporting information

S1 File Measurement protocol. (PDF)

S2 File Analysis of eye movement data. (PDF)

S1 Table Descriptive and reproducibility results of the fixation task. BCEA: bivariate contour ellipse area, IQR: interquartile range, SE: standard error of the estimate, SWJ: square wave jerk, deg: degrees, s: seconds, ms: milliseconds, nr: number, SD: standard deviation, ICC: intra-class correlation coefficient, CI: confidence interval, CV: coefficient of variation, CR: coefficient of repeatability. For every parameters, the upper row represents the first set of measurements, the lower row the second set of measurements.
(PDF)

S2 Table Descriptive and reproducibility results of the pro-saccadic task. VDI: versional dysconjugacy index, FPG: first-pass gain, AUC: area under the curve, deg: degrees, s: seconds, ms: milliseconds, SD: standard deviation, ICC: intra-class correlation coefficient, CI: confidence interval, CV: coefficient of variation, CR: coefficient of repeatability. For every parameters, the upper row represents the first set of measurements, the lower row the second set of measurements.
(PDF)

S3 Table Descriptive and reproducibility results of the anti-saccadic task. AS: anti-saccades, PS: pro-saccades, FEP: final eye position, deg: degrees, s: seconds, ms: milliseconds, SD: standard deviation, ICC: intra-class correlation coefficient, CI: confidence interval, CV: coefficient of variation, CR: coefficient of repeatability. For every parameters, the upper row represents the first set of measurements, the lower row the second set of measurements.
(PDF)

S4 Table Descriptive and reproducibility results of the express saccadic task. deg: degrees, s: seconds, ms: milliseconds, SD: standard deviation, ICC: intra-class correlation coefficient, CI: confidence interval, CV: coefficient of variation, CR: coefficient of repeatability. For every parameters, the upper row represents the first set of measurements, the lower row the second set of measurements.
(PDF)

S5 Table Descriptive and reproducibility results of the double-step saccadic task. FS: first saccade, SS: second saccade, DS: double-step saccade, deg: degrees, s: seconds, ms: milliseconds, SD: standard deviation, ICC: intra-class correlation coefficient, CI: confidence interval, CV: coefficient of variation, CR: coefficient of repeatability. For every parameters, the upper row represents the first set of measurements, the lower row the second set of measurements.
(PDF)

S6 Table Descriptive and reproducibility results of the repeated pro-saccadic task. VDI: versional dysconjugacy index, deg: degrees, s: seconds, ms: milliseconds, SD: standard deviation, ICC: intra-class correlation coefficient, CI: confidence interval, CV: coefficient of variation, CR: coefficient of repeatability. For every parameters, the upper row represents the first set of measurements, the lower row the second set of measurements.
(PDF)

S7 Table Descriptive results of all tasks of center two. BCEA: bivariate contour ellipse area, IQR: interquartile range, SE: standard error of the estimate, SWJ: square wave jerk, VDI: versional dysconjugacy index, FPG: first-pass gain, AUC: area under the curve, AS: anti-saccades, PS: pro-saccades, FEP: final eye position, FS: first saccade, SS: second saccade, DS: double-step saccade, deg: degrees, s: seconds, ms: milliseconds, nr: number, SD: standard deviation.
(PDF)

S1 Fig Welch power spectrum of raw data of one fixation trial. Data of one subject measured in both centers. The red line represents the data from center one at the original sampling frequency of 1000 Hz, the blue line the same data downsampled to 500 Hz and the green line the data of center two sampled at 500 Hz.
(TIF)

S2 Fig Horizontal gaze, velocity and acceleration signal. Signal (filtered data of center one) of a leftward 8 degrees saccade, the same saccade is shown at 1000 Hz (left) and downsampled to 500 Hz (right).
(TIF)
