# Supplementary material for: A standardized protocol for quantification of saccadic eye movements: DEMoNS
Source: PLoS One. 2018 Jul 16;13(7):e0200695. doi: 10.1371/journal.pone.0200695 (PMC6047815; doi:10.1371/journal.pone.0200695)
Supplement: S1 File — (PDF) [file pone.0200695.s001.pdf]

S1 File - Measurement protocol

In the following section, a description of every task and subject instructions is included.

1. Fixations

Visual fixation is highly dependent on the neural integrator. The neuronal integrator represents a distributed network of neurons in the brainstem and cerebellum. Furthermore, the omnipause neurons (in the nucleus raphe interpositus in the pons) and some focal cerebral areas are involved [1-4].

The protocol contains two trials of five fixations, of eight seconds each, at the center or an eccentric location (15 degrees of visual angle left or right or 10 degrees of visual angle up or down from the center). The subject instruction is as follows: **"**Look at the circle and follow it when it jumps to another position. The dot will sometimes remain at one location for quite a long time."

2. Horizontal pro-saccades

The possibility to make saccades in different directions is primarily based on the control of the six extra-ocular muscles by the lower motor neurons. The three ocular motor nuclei (figure 1) in the brainstem have internuclear connections (through the medial longitudinal fasciculus, MLF) and are activated by premotor burst neurons. For horizontal movements, these are located in the paramedian pontine reticular formation (PPRF). The accuracy of a saccade relies on areas in the cerebellum (dorsal vermis and fastigial nucleus), and the superior colliculus is an important structure for initiation of the saccades [1-3].

The protocol contains five trials of 12 pro-saccades from the center to an eccentric location, either 8 or 15 degrees of visual angle left or right of the center. Each location is addressed trice in each trial, in a random order. The eccentric target appears for 1.5 seconds, after a fixation period at the center with a random duration between 1.0 and 3.5 seconds. The first trial is preceded by a practice trial, containing four pro-saccades. The subject instruction is as follows: **"**Look at the circle and follow it when it jumps to another position. The target will only be displaced horizontally."

3. Anti-saccades

Anti-saccades are saccades directed opposite to a target location. They are a measure of cognitive

function, primarily dependent upon the integrity of frontal and parietal region. The two main areas involved are the dorsolateral prefrontal cortex, mainly responsible for inhibition of the reflexive response, and the frontal eye field, mainly responsible for (fast) initiation of the correct response. The cingulate eye field, supplementary eye field and posterior parietal cortex are additional areas involved [2, 3, 5, 6].

The protocol contains four trials of 10 anti-saccades from the center to an eccentric location . The eccentric location is 8 degrees of visual angle left or right of the center, both addressed five times in every trial, in a random order. The eccentric target appears for 1.5 seconds, after a fixation period at the center with a random duration between 1.0 and 3.5 seconds. The subject has to make a saccade in the opposite direction. The target will appear at the side of fixation for 750 ms, and at that point the subject has to fixate it. The first trial is preceded by an example trial (in which arrows are used for instructions) and a practice trial, both containing four anti-saccades.

The subject instruction is a follows**: “**Look at the circle, when it jumps to the left or right, look in the exact opposite position. Look back at the target when it appears on this side. Note this task is different to the one you have just performed. I will show you an example first.”

4. Express saccades

Express saccades are short-latency saccades, after appearance of a stimulus with a gap period. The possibility to make these saccades is mainly dependent on function of the superior colliculus (figure 1) [2, 3].

The protocol contains three trials of 10 express saccades from the center to an eccentric location, either 8 degrees of visual angle left or right from the center. Each location is addressed five times in each trial, in a random order. After a fixation period at the center with a random duration between 1.0 and 3.5 seconds, the central target will disappear and after a time interval of 200 ms, it appears at an eccentric location. The first trial is preceded by a practice trial, containing four express saccades.

The subject instruction is as follows: **“**Look at the circle and follow it when it jumps to another position. The target will only be displaced horizontally.”

5. Double-step saccades

Double-step saccades are saccades towards two stimuli that are quickly presented in succession at

two different locations, at which retinal-spatial dissonance is created (both targets have disappeared before the first saccade). Correct execution of the second saccade requires knowledge about the first saccade's metrics, without visual feedback. The mechanism probably used for this purpose is the corollary discharge, which gives an internal copy of an impending motor command. Important for this mechanism are the posterior parietal cortex, parietal eye field and central thalamus (figure 1). Additionally the supplementary eye field, frontal eye field and superior colliculus are involved [2, 3, 7-9].

The protocol contains five trials of 12 double-step saccades from the center to four eccentric locations. The location of the first target is either 8 degrees of visual angle left or right from the center, the location of the second target is always 8 degrees from the first target, oriented up of down and 45 degrees more to the center than the first target (see figure 3). Each combination of locations is addressed trice in each trial, in a random order. Target one and two will appear in sequence, visible for 67 ms each, after a fixation period at the center with a random duration between 1.0 and 3.5 seconds. The subject has to make saccades to the two locations in order of appearance. The target will re-appear for 750 ms at the second location after an interval of 1.0 second, the subject has to fixate it. The first trial is preceded by a practice trial, containing four double-step saccades.

The subject instruction is as follows**:** "Look at the circle. It will quickly jump to two different locations, follow it quickly in the order of their appearance. It will reappear at the last location, look at it."

The instructions between the trials is as follows: "Keep looking at the dot in the order of its appearance".

6. Repeated pro-saccades

Repeating the saccades task can test ocular motor fatigability and could probably provide an objective test to discriminate between fatigued and non-fatigued patients, related to subjective fatigue displayed on self-rated questionnaires [10, 11].

The protocol contains three trials of 10 pro-saccades from the center to an eccentric location, either 8 degrees left or right from the center. Each location is addressed five times in each trial, in a random order. The eccentric target appears for 1.5 seconds, after a fixation period at the center with a random duration between 1.0 and 3.5 seconds. The first trial is preceded by a practice trial, containing four pro-saccades.

The subject instructions is as follows**: “**Look at the dot and follow it when it jumps to another position. The target will only be displaced horizontally.”

S1 File - References

1. Frohman EM, Frohman TC, Zee DS, McColl R, Galetta S. The neuro-ophthalmology of multiple sclerosis. The Lancet Neurology. 2005;4(2):111-21.
2. Leigh RJ, Zee DS. The neurology of eye movements. 5 ed. Oxford: Oxford University Press; 2015.
3. Leigh RJ, Kennard C. Using saccades as a research tool in the clinical neurosciences. Brain. 2004;127:460-77.
4. Karatas M. Internuclear and supranuclear disorders of eye movements: clinical features and causes. Eur J Neurol. 2009;16(12):1265-77.
5. Hutton SB, Ettinger U. The antisaccade task as a research tool in psychopathology: a critical review. Psychophysiology. 2006;43(3):302-13.
6. Pierrot-Deseillignya C, Mileab D, Müri RM. Eye movement control by the cerebral cortex. Curr Opin Neurol. 2004;17:17–25.
7. Heide W, Blankenburg M, Zimmermann E, Kömpf D. Cortical control of double-step saccades: implications for spatial orientation. Ann Neurol. 1995;38:739-48.
8. Sommer MA, Wurtz RH. A pathway in primate brain for internal monitoring of movements. Science. 2002;296:1480-2.
9. Buonocore A, Melcher D. Disrupting saccadic updating: visual interference prior to the first saccade elicits spatial errors in the secondary saccade in a double-step task. Exp Brain Res. 2015;233(6):1893-905.
10. Matta M, Leigh RJ, Pugliatti M, Aiello I, Serra A. Using fast eye movements to study fatigue in multiple sclerosis. Neurology. 2009;73:798-804.
11. Finke C, Pech LM, Sommer C, Schlichting J, Stricker S, Endres M, et al. Dynamics of saccade parameters in multiple sclerosis patients with fatigue. J Neurol. 2012;259(12):2656-63.
