# Supplementary material for: A standardized protocol for quantification of saccadic eye movements: DEMoNS
Source: PLoS One. 2018 Jul 16;13(7):e0200695. doi: 10.1371/journal.pone.0200695 (PMC6047815; doi:10.1371/journal.pone.0200695)
Supplement: S2 File — (PDF) [file pone.0200695.s002.pdf]

S2 File – Analysis of eye movement data

This section summarizes the data analysis protocol.

Pre-processing

Noise and glitches in the signal were removed by filtering the raw eye position signal using a maximally flat 2nd order low-pass filter without delay. The cut-off frequency was set at 10% of the sampling frequency. To further remove noise, and prepare for the velocity estimate, the output of this filter is convoluted with a 9-sample wide edge enhancing smoothing kernel.

The increment of the smoothed filtered eye position multiplied by the sampling frequency is used to determine the velocity (in pixels per second) of the eye. The velocity is then converted to degrees of visual angle per second, by dividing every sample with the corresponding pixels per degree (PPD) for that sample. The increment in the velocity signal multiplied by the sampling frequency is used to determine the acceleration (in degrees per second per second) of the eye.

In order to eliminate blink artefacts, removal of samples was performed between 0.10 seconds before, and 0.25 seconds after a blink.

Saccadic detection

Saccadic identification was performed in five different steps, which are summarized in Table 1. The algorithm is based on validated algorithms described in literature [1-3].

Approximate saccade interval were identified using an adaptive acceleration threshold, based on the 99% confidence interval of the distribution of the acceleration signal in horizontal and vertical direction. The advantage of an adaptive threshold is the ability to take differences between participants and trials into account. The acceleration of saccades is highly discriminative from other type of eye movements, while the velocity range of (slow) saccades can overlap with that of nystagmus, pursuit and ocular drift movements. Samples were included in the approximate saccade interval if either the X or Y acceleration signal was above their own threshold.

Next, the intervals were merged if they occurred closer than 20 ms. By this, also possible post-saccadic oscillations (PSO) were included in the interval. Subsequently, the exact saccadic onset and

offset were detected, by checking consecutive samples in backward and forward direction respectively, starting at the sample with the highest velocity. At this starting sample, the main direction of the saccade was determined, by determining the sample-to-sample direction at this point. Three criteria were used for both the onset and offset, (1) a sample-to-sample direction deviation from the main direction of more than 60 degrees, (2) a sample-to-sample directional change between two adjacent samples of more than 20 degrees per ms of sample duration (e.g. 20 degrees at a sample frequency of 1000 Hz, 40 degrees at a sample frequency of 500 Hz) and (3) a speed of <5 degrees per second. If a sample satisfied one of the three criteria, the sample before or after (closest to the starting sample) this sample was considered as the start or end of the saccade. The different types of thresholds were chosen by taking in mind physical properties of saccades, the fast movement and the ballistic behaviour of the movement.

To further eliminate possible noise included as saccades, the saccades had to be at least 0.15 degrees in amplitude and eight ms in duration to be valid. Finally, the main saccade of a saccade interval (saccades which are closer than 20 ms apart) was determined, by taking the saccade with the highest peak velocity. Subsequent saccades in the interval were classified as PSO’s and were not taken into account for further analysis.

Parameters fixation

In the fixation task, the period between one second after a target movement to a fixation location and the target movement back to the center, were considered as fixation periods. This resulted in a maximum of 10 fixation periods of seven seconds, at five different locations, for every measurement. For this paper, the mean of the five different target positions was calculated for every parameter, providing an overall impression of fixation performance in different directions.

In the fixation periods, the stability of the fixation was calculated from non-saccadic samples, based on the gaze and vergence signals. The fixation stability was expressed as the mean and SD of the horizontal and vertical gaze position and vergence, mean and SD of horizontal and vertical velocity, median and IQR (interquartile range) of total velocity, and mean and SD of total acceleration. Furthermore the Bivariate Contour Ellipse Area (BCEA, see figure 4) was calculated for the gaze position and vergence, which is the area of a bivariate ellipse encompassing a given proportion the

highest density samples [4, 5]. This can be considered as a composite of the SD of horizontal and vertical dimension, taken into account the correlation between the horizontal and vertical gaze position or vergence . The formula is as follows:

$$BCEA=2\chi^2\pi\sigma_h\sigma_v(1-\rho)^{0.5}$$

The σ_h and σ_v in this formula are the standard deviation of the horizontal and vertical gaze positions or vergence respectively, ρ represents the product-moment correlation of these two components, and χ^2 is a chi-square variable. In line with common practice, χ^2 was set to 2.291 so that the BCEA compasses 68% of the highest density points (corresponding to the area within one SD from the mean in a normal distribution).

Next, a linear fit (regression line) was drawn through all the gaze position and vergence fixation samples of one target position (max. 14 sec), using the linear least squares method. The regression coefficient (converted to degrees per second) and the standard error of the estimate (reflecting the mean deviation of the gaze or vergence from the regression line) were calculated.

In addition, the number of saccadic intrusions during the fixation periods were counted, and grouped into square wave jerks (SWJs), macrosquare wave jerks (MSWJs), microsaccades and large intrusive saccades. SWJs were defined as saccades with an amplitude between 0.1 and 4.0 degrees, followed between 50 ms and 400 ms by another saccade with an amplitude comparable to the first saccade (<0.75 degrees difference) and in the opposite direction (angle between the two directions between 90 and 270 degrees). MSWJs were defined similar, but with an amplitude >4.0 degrees [6].

All other saccades (not part of SWJ's or MSWJ's) during the fixation periods were defined as microsaccades if the amplitude was below 2 degrees, and as a large intrusive saccade if the amplitude was 2 degrees or above.

The number of all saccadic intrusions was expressed as the mean number of saccadic intrusions per second. The mean amplitude of SWJs + MSWJs and other saccades was calculated, furthermore the mean time between the end of the first and start of the second saccade of a SWJ or MSWJ (intra-SWJ interval) was determined.

Parameters (repeated) pro-saccades and express saccades

In the pro-saccadic, express saccadic and repeated pro-saccadic task, automated detection of the correct main centrifugal saccade was based on a few selection criteria. The saccade should start at least 50 ms after the target movement (to exclude anticipatory responses), with a maximum of 1000 ms, start within a visual angle of four degrees to the left or right of the center, has to go in the right direction (left or right) and should have an amplitude of at least 7.5 or 4 degrees (respectively to 15 and 8 degrees target movement).

The peak velocity, peak acceleration, gain and latency of all included saccades were calculated. The gain was determined by dividing the amplitude of the main saccade by the mean amplitude of the fixation after corrective saccade(s), instead of dividing it by the absolute position of the target position. This was performed to minimize the effects of inaccuracies of the system in measuring the absolute eye position. Gain values higher than one reflect hypermetric saccades (overshoot of the target) and values below one hypometric saccades (undershoot of the target). Furthermore, the first pass gain (FPG) was calculated, which is the gain at the time point where the abducting eye first reaches the fixation position after the saccade.[7] Next the area under the curve of the saccadic trajectory of the horizontal eye position (AUC) was calculated, which is the sum of the distance between the horizontal amplitude and the horizontal start position of the saccade.

For all the saccades separately, saccade pair ratios were determined, by dividing the abducting eye value by the adducting eye value. This ratio is referred to as the versional dysconjugacy index (VDI) [8]. This was calculated for peak velocity, peak acceleration, FPG and AUC.

Only binocular saccades were taken into account, and for all the parameters (apart from the VDIs) the mean of the left and right eye was taken.

Parameters anti-saccades

In the anti-saccadic task, the main saccade should start between 50 and 1000 ms after the target movement, within a visual angle of four degrees left or right of the center and with an amplitude of at least two degrees. If the direction of this saccade was in the opposite direction from the target movement, this was classified as a correct anti-saccade, otherwise as an incorrect pro-saccade. After an incorrect pro-saccade, the first saccade crossing the center was considered as the main corrective saccade. The proportion of errors (number of errors divided by the number of included saccades),

latency of the main saccade and the corrective saccade, and the gain and error of both the correct anti-saccades as the final eye position (FEP, position before refixation of the eccentric target) were calculated.

Parameters double-step saccades

In the double-step task the first saccade should start between 50 and 1000 ms after the target movement, within a visual angle of four degrees left or right of the center and with an amplitude of at least two degrees. If this saccade was in the correct direction (deviating not more than 45 degrees from the direction of the first target movement) and ending within a visual angle of four degrees of the first target position, this was considered as a correct first saccade.

The second saccade should start after the end of the first saccade and before the centripetal target movement, within a visual angle of four degrees of the location of the first target, with an amplitude of at least two degrees and ending more than four degrees from the first target location. This last criterion was added to prevent inclusion of corrective saccades at the first target location. If this saccade was in the right direction (deviating not more than 45 degrees from the direction of the second target movement) and ending within a visual angle of four degrees from the second target location and before the re-appearance of the second target, this was considered a correct second saccade.

The peak velocity, acceleration and direction difference of first and second saccade, latency and amplitude of first saccade, intersaccadic interval and gain of second saccade were calculated. The FEP was defined as the mean eye position after the last (corrective) saccade, before refixation of the second target. The gain, X and Y errors (negative values of the errors represent undershoot) and total absolute error (taking both X and Y error into account) of the FEP were calculated.

Furthermore, the proportion (of the total number of saccades) of correct double-step saccades, acceptable double-step saccades (adding the proportion of first and/or second saccades which were correctly directed, but not ending within 4 degrees of the target) were calculated. In addition, the proportion of contraversive shifted double-step saccades (second saccade directed as if starting from fixation point, in the direction of second target location) and late double-step saccades (second saccade is correct, but not ending before reappearance of the second target) were determined. Finally, the proportion of first saccades that are directed to the second target location was calculated.

S2 File - References

1. Engbert R, Kliegl R. Microsaccades uncover the orientation of covert attention. Vision Research. 2003;43(9):1035-45.

2. Larsson L, Nyström M, Stridh M. Detection of saccades and postsaccadic oscillations in the presence of smooth pursuit. IEEE Transactions on biomedical engineering. 2013;60(9):2484-93.

3. Nystrom M, Holmqvist K. An adaptive algorithm for fixation, saccade, and glissade detection in eyetracking data. Behav Res Methods. 2010;42(1):188-204.

4. Steinman RM. Effect of target size, luminance, and color on monocular fixation. Journal of the optical society of America. 1965;55(9):1158-65.

5. Castet E, Crossland M. Quantifying eye stability during a fixation task: a review of definitions and methods. Seeing Perceiving. 2012;25(5):449-69.

6. Leigh RJ, Zee DS. The neurology of eye movements. 5 ed. Oxford: Oxford University Press; 2015.

7. Frohman EM, O'Suilleabhain P, Dewey RB, Frohman TC, Kramer PD. A new measure of dysconjugacy in INO: the first-pass amplitude. Journal of the Neurological Sciences. 2003;210(1-2):65-71.

8. Ventre J, Vighetto A, Bailly G, Prablanc C. Saccade metrics in multiple sclerosis: versional velocity disconjugacy as the best clue? Journal of the Neurological Sciences. 1991;102:144-9.
