# Supplementary material for: A standardized protocol for quantification of saccadic eye movements: DEMoNS
Source: PLoS One. 2018 Jul 16;13(7):e0200695. doi: 10.1371/journal.pone.0200695 (PMC6047815; doi:10.1371/journal.pone.0200695)
Supplement: S1 Table — BCEA: bivariate contour ellipse area, IQR: interquartile range, SE: standard error of the estimate, SWJ: square wave jerk, deg: degrees, s: seconds, ms: milliseconds, nr: number, SD: standard deviation, ICC: intra-class correlation coefficient, CI: confidence interval, CV: coefficient of variation, CR: coefficient of repeatability. For every parameters, the upper row represents the first set of measurements, the lower row the second set of measurements. (PDF) [file pone.0200695.s003.pdf]

S1 Table. Descriptive and reproducibility results of the fixation task

Parameter	Mean	SD	Range	ICC (95% CI)	CR	CV (%)
SD X gaze (deg)	0.18	0.07	0.10 – 0.37	0.70 (0.37 – 0.87)	0.06	11.4
	0.18	0.05	0.11 – 0.30			
SD Y gaze (deg)	0.20	0.06	0.11 – 0.31	0.79 (0.53 – 0.91)	0.07	13.2
	0.21	0.08	0.11 – 0.36			
BCEA gaze (deg^2)	0.21	0.10	0.06 – 0.42	0.71 (0.39 – 0.88)	0.10	17.1
	0.20	0.10	0.07 – 0.41			
SD X vergence (deg)	0.21	0.08	0.09 – 0.44	0.85 (0.62 – 0.94)	0.07	12.2
	0.23	0.08	0.11 – 0.41			
SD Y vergence (deg)	0.21	0.07	0.13 – 0.47	0.67 (0.33 – 0.86)	0.09	15.8
	0.22	0.09	0.13 – 0.52			
BCEA vergence (deg^2)	0.28	0.16	0.09 – 0.44	0.82 (0.58 – 0.93)	0.15	20.6
	0.32	0.17	0.10 – 0.70			
Mean velocity X (deg/s)	-0.01	0.04	-0.13 – 0.04	0.66 (0.31 – 0.85)	0.06	N/A
	-0.01	0.04	-0.15 – 0.05			
SD velocity X (deg/s)	2.70	0.81	1.76 – 5.29	0.78 (0.52 – 0.91)	0.56	6.7
	2.64	0.56	1.72 – 3.78			
Mean velocity Y (deg)	0.01	0.07	-0.11 – 0.14	0.96 (0.89 – 0.98)	0.03	N/A
	0.00	0.07	-0.12 – 0.13			
SD velocity Y (deg)	2.97	0.83	1.92 – 5.32	0.94 (0.85 – 0.98)	0.42	5.1
	3.04	0.80	1.74 – 5.18			
Median velocity total (deg/s)	3.00	0.87	1.97 – 5.92	0.89 (0.75 – 0.96)	0.44	4.8
	2.98	0.66	1.85 – 4.67			
IQR velocity total (deg/s)	2.48	0.66	1.64 – 4.64	0.89 (0.74 – 0.96)	0.39	5.3
	2.48	0.53	1.53 – 3.81			
Linear fit coefficient X gaze (deg/s)	-0.01	0.06	-0.14 – 0.16	0.63 (0.27 – 0.84)	0.07	N/A
	0.00	0.05	-0.14 – 0.10			
SE linear fit X gaze (deg)	0.15	0.06	0.08 – 0.35	0.53 (0.11 – 0.79)	0.05	10.5
	0.15	0.03	0.08 – 0.20			
Linear fit coefficient Y gaze (deg/s)	0.01	0.07	-0.12 – 0.10	0.90 (0.77 – 0.96)	0.05	N/A
	0.00	0.07	-0.13 – 0.14			

SE linear fit Y gaze (deg)	0.15<br>0.16	0.03<br>0.05	0.09 – 0.19<br>0.09 – 0.27	0.30 (-0.15 – 0.65)	0.07	15.8
Linear fit coefficient X vergence (deg/s)	0.04<br>0.05	0.07<br>0.06	-0.10 – 0.18<br>-0.02 – 0.17	0.69 (0.37 – 0.87)	0.07	N/A
SE linear fit X vergence (deg)	0.21<br>0.22	0.07<br>0.06	0.09 – 0.34<br>0.11 – 0.33	0.67 (0.33 – 0.86)	0.08	13.7
Linear fit coefficient Y vergence (deg/s)	0.01<br>0.02	0.04<br>0.04	-0.08 – 0.09<br>-0.05 – 0.08	0.15 (-0.32 – 0.56)	0.08	N/A
SE linear fit Y vergence (deg)	0.21<br>0.24	0.07<br>0.10	0.14 – 0.43<br>0.14 – 0.56	0.49 (0.08 – 0.76)	0.13	19.1
SWJ >4 deg (nr/s)	0<br>0	0<br>0	0 – 0<br>0 – 0.01	N/A	0.00	N/A
SWJ <4 deg (nr/s)	0.19<br>0.21	0.12<br>0.17	0.00 – 0.50<br>0.00 – 0.65	0.68 (0.34 – 0.86)	0.18	32.0
Mean amplitude SWJ (deg)	0.52<br>0.53	0.37<br>0.40	0.24 – 1.77<br>0.18 – 1.94	0.92 (0.80 – 0.97)	0.22	16.4
Saccades >2 deg (nr/s)	0.01<br>0.01	0.02<br>0.02	0.00 – 0.06<br>0.00 – 0.05	0.74 (0.45 – 0.89)	0.02	87.7
Saccades <2 deg (nr/s)	0.60<br>0.64	0.34<br>0.32	0.26 – 1.45<br>0.20 – 1.36	0.84 (0.63 – 0.93)	0.28	16.8
Mean amplitude saccades (deg)	0.46<br>0.41	0.30<br>0.21	0.22 – 1.45<br>0.24 – 1.05	0.86 (0.68 – 0.94)	0.16	11.4
Mean amplitude SWJ + saccades (deg)	0.48<br>0.45	0.30<br>0.28	0.23 – 1.48<br>0.25 – 1.49	0.95 (0.88 – 0.98)	0.12	10.2
Mean duration intra-SWJ interval (ms)	231<br>230	35<br>46	160 – 282<br>107 – 279	0.71 (0.37 – 0.88)	45	7.9
