# Supplementary material for: A standardized protocol for quantification of saccadic eye movements: DEMoNS
Source: PLoS One. 2018 Jul 16;13(7):e0200695. doi: 10.1371/journal.pone.0200695 (PMC6047815; doi:10.1371/journal.pone.0200695)
Supplement: S2 Table — VDI: versional dysconjugacy index, FPG: first-pass gain, AUC: area under the curve, deg: degrees, s: seconds, ms: milliseconds, SD: standard deviation, ICC: intra-class correlation coefficient, CI: confidence interval, CV: coefficient of variation, CR: coefficient of repeatability. For every parameters, the upper row represents the first set of measurements, the lower row the second set of measurements. (PDF) [file pone.0200695.s004.pdf]

S2 Table. Descriptive and reproducibility results of the pro-saccadic task

Parameter	Mean	SD	Range	ICC (95% CI)	CR	CV (%)
Peak velocity (deg/s)	370	46	279 – 449	0.94 (0.84 – 0.97)	25	2.4
	376	45	304 – 467			
Peak velocity 15 deg (deg/s)	397	49	299 – 480	0.93 (0.82 – 0.97)	31	2.7
	405	50	326 - 509			
Peak velocity 8 deg (deg/s)	342	44	260 – 420	0.92 (0.81 – 0.97)	26	2.8
	347	43	281 - 429			
Peak acceleration (deg/s^2)	34704	4989	26269 – 42116	0.93 (0.83 – 0.97)	2663	2.7
	35281	5226	27960 – 46059			
Peak acceleration 15 deg (deg/s^2)	36235	5283	28525 – 45198	0.92 (0.82 – 0.97)	2873	2.8
	36965	5676	28827 – 49601			
Peak acceleration 8 deg (deg/s^2)	33161	4791	24088 – 40276	0.92 (0.77 – 0.97)	2944	3.1
	33633	4883	25525 – 42888			
Latency (ms)	186	22	152 – 241	0.90 (0.77 – 0.96)	13	2.6
	184	23	150 - 238			
Latency 15 deg (ms)	197	24	158 – 255	0.90 (0.76 – 0.96)	15	2.8
	194	27	159 – 251			
Latency 8 deg (ms)	176	21	145 – 227	0.89 (0.74 – 0.96)	16	3.3
	174	21	141 – 226			
Gain	0.96	0.04	0.89 – 1.03	0.77 (0.52 – 0.91)	0.04	1.5
	0.97	0.03	0.89 – 1.04			
Gain 15 deg	0.93	0.04	0.82 – 0.99	0.77 (0.50 – 0.90)	0.04	1.6
	0.94	0.03	0.84 – 0.99			
Gain 8 deg	0.98	0.05	0.90 – 1.08	0.77 (0.49 – 0.90)	0.06	2.0
	1.00	0.05	0.93 – 1.10			
Peak velocity / amplitude	36.0	4.2	28.5 – 41.3	0.95 (0.87 – 0.98)	2.1	2.1
	36.4	4.1	31.1 – 44.7			
Peak velocity / amplitude 15 deg	29.3	3.6	22.6 – 33.6	0.95 (0.88 – 0.98)	1.7	2.1
	29.5	3.4	24.2 – 35.5			
Peak velocity / amplitude 8 deg	42.8	5.0	34.0 – 50.1	0.95 (0.87 – 0.98)	2.5	2.1
	43.2	5.1	36.1 – 53.1			

VDI peak velocity	1.07<br>1.07	0.08<br>0.08	0.91 – 1.19<br>0.93 – 1.24	0.95 (0.87 – 0.98)	0.04	1.4
VDI peak velocity 15 deg	1.07<br>1.08	0.08<br>0.08	0.91 – 1.20<br>0.95 – 1.24	0.93 (0.84 – 0.97)	0.04	1.5
VDI peak velocity 8 deg	1.07<br>1.07	0.07<br>0.08	0.91 – 1.18<br>0.91 – 1.24	0.95 (0.88 – 0.98)	0.04	1.4
VDI peak velocity left	1.08<br>1.08	0.08<br>0.09	0.95 – 1.22<br>0.96 – 1.29	0.95 (0.87 – 0.98)	0.04	1.5
VDI peak velocity right	1.07<br>1.06	0.08<br>0.08	0.87 – 1.18<br>0.91 – 1.19	0.87 (0.70 – 0.95)	0.07	2.3
VDI peak acceleration	1.11<br>1.11	0.11<br>0.12	0.90 – 1.29<br>0.91 – 1.30	0.95 (0.88 – 0.98)	0.05	1.8
VDI peak acceleration 15 deg	1.11<br>1.11	0.10<br>0.11	0.91 – 1.29<br>0.93 – 1.30	0.91 (0.79 – 0.97)	0.07	2.3
VDI peak acceleration 8 deg	1.10<br>1.10	0.11<br>0.12	0.89 – 1.28<br>0.90 – 1.29	0.96 (0.90 – 0.98)	0.05	1.7
VDI peak acceleration left	1.12<br>1.12	0.13<br>0.15	0.90 – 1.36<br>0.88 – 1.40	0.97 (0.92 – 0.99)	0.06	1.9
VDI peak acceleration right	1.09<br>1.09	0.11<br>0.11	0.90 – 1.26<br>0.93 – 1.28	0.82 (0.59 – 0.93)	0.11	3.5
VDI FPG	1.03<br>1.03	0.03<br>0.04	0.97 – 1.10<br>0.96 – 1.10	0.93 (0.82 – 0.98)	0.02	0.7
VDI FPG 15 deg	1.01<br>1.02	0.02<br>0.02	0.97 – 1.05<br>0.98 – 1.07	0.92 (0.80 – 0.97)	0.01	0.4
VDI FPG 8 deg	1.04<br>1.04	0.04<br>0.05	0.96 – 1.14<br>0.95 – 1.13	0.88 (0.72 – 0.95)	0.03	1.2
VDI FPG left	1.03<br>1.03	0.04<br>0.04	0.98 – 1.13<br>0.97 – 1.14	0.85 (0.67 – 0.94)	0.03	1.1
VDI FPG right	1.03<br>1.03	0.03<br>0.04	0.96 – 1.09<br>0.96 – 1.11	0.91 (0.79 – 0.97)	0.02	0.8
VDI AUC	1.08<br>1.08	0.06<br>0.06	0.95 – 1.18<br>0.95 – 1.21	0.95 (0.87 – 0.98)	0.03	1.0
VDI AUC 15 deg	1.07<br>1.07	0.06<br>0.05	0.94 – 1.15<br>0.96 – 1.17	0.92 (0.80 – 0.97)	0.03	1.1

VDI AUC 8 deg	1.09<br>1.09	0.07<br>0.07	0.95 – 1.21<br>0.95 – 1.24	0.96 (0.90 – 0.98)	0.03	1.0
VDI AUC left	1.08<br>1.09	0.07<br>0.07	0.97 – 1.22<br>1.00 – 1.23	0.92 (0.80 – 0.97)	0.05	1.6
VDI AUC right	1.07<br>1.07	0.06<br>0.06	0.91 – 1.14<br>0.91 – 1.18	0.87 (0.69 – 0.95)	0.05	1.6
VDI peak velocity / amplitude	1.05<br>1.05	0.06<br>0.06	0.96 – 1.16<br>0.97 – 1.19	0.95 (0.88 – 0.98)	0.03	1.0
VDI peak velocity / amplitude 15 deg	1.05<br>1.05	0.07<br>0.07	0.95 – 1.18<br>0.97 – 1.21	0.94 (0.86 – 0.98)	0.04	1.3
VDI peak velocity / amplitude 8 deg	1.05<br>1.05	0.05<br>0.06	0.97 – 1.14<br>0.97 – 1.18	0.93 (0.83 – 0.97)	0.03	1.1
VDI peak velocity / amplitude left	1.06<br>1.06	0.07<br>0.06	0.97 – 1.17<br>0.97 – 1.21	0.92 (0.81 – 0.97)	0.04	1.3
VDI peak velocity / amplitude right	1.04<br>1.04	0.06<br>0.07	0.91 – 1.14<br>0.95 – 1.17	0.92 (0.80 – 0.97)	0.04	1.5
