# Supplementary material for: A standardized protocol for quantification of saccadic eye movements: DEMoNS
Source: PLoS One. 2018 Jul 16;13(7):e0200695. doi: 10.1371/journal.pone.0200695 (PMC6047815; doi:10.1371/journal.pone.0200695)
Supplement: S3 Table — AS: anti-saccades, PS: pro-saccades, FEP: final eye position, deg: degrees, s: seconds, ms: milliseconds, SD: standard deviation, ICC: intra-class correlation coefficient, CI: confidence interval, CV: coefficient of variation, CR: coefficient of repeatability. For every parameters, the upper row represents the first set of measurements, the lower row the second set of measurements. (PDF) [file pone.0200695.s005.pdf]

S3 Table. Descriptive and reproducibility results of the anti-saccadic task

Parameter	Mean	SD	Range	ICC (95% CI)	CR	CV (%)
Peak velocity AS (deg/s)	341<br>343	57<br>59	212 – 455<br>230 – 444	0.86 (0.68 – 0.95)	42	4.3
Peak acceleration AS (deg/s^2)	33961<br>34028	5562<br>5655	22061 – 40086<br>24341 – 45265	0.85 (0.64 -0.94)	4654	4.8
Latency (ms)	286<br>280	52<br>50	197 – 398<br>202 – 398	0.83 (0.62 – 0.93)	43	5.3
Latency AS (ms)	331<br>313	92<br>71	216 – 580<br>211 – 519	0.83 (0.60 – 0.93)	65	6.8
Latency PS (ms)	220<br>207	44<br>47	157 – 300<br>154 – 317	0.55 (0.14 – 0.80)	67	10.9
Gain AS	1.11<br>1.05	0.29<br>0.21	0.60 – 1.80<br>0.72 – 1.46	0.54 (0.14 – 0.79)	0.25	7.9
X error AS (deg)	0.54<br>0.32	1.86<br>1.58	-3.15 – 3.34<br>-2.18 – 3.06	0.76 (0.49 – 0.90)	1.59	N/A
Proportion errors	0.30<br>0.25	0.21<br>0.18	0.08 – 0.85<br>0.00 – 0.77	0.78 (0.52 – 0.91)	0.22	38.7
Latency correction PS (ms)	205<br>184	83<br>65	95 – 387<br>64 – 336	0.54 (0.14 – 0.80)	113	22.1
Gain FEP	1.08<br>1.04	0.19<br>0.10	0.75 – 1.62<br>0.90 – 1.22	0.04 (-0.42 – 0.48)	0.23	7.5
X error FEP (deg)	0.47<br>0.23	1.13<br>0.73	-1.68 – 2.88<br>-0.81 – 1.72	0.20 (-0.27 – 0.59)	1.48	N/A
