# Supplementary material for: A standardized protocol for quantification of saccadic eye movements: DEMoNS
Source: PLoS One. 2018 Jul 16;13(7):e0200695. doi: 10.1371/journal.pone.0200695 (PMC6047815; doi:10.1371/journal.pone.0200695)
Supplement: S4 Table — deg: degrees, s: seconds, ms: milliseconds, SD: standard deviation, ICC: intra-class correlation coefficient, CI: confidence interval, CV: coefficient of variation, CR: coefficient of repeatability. For every parameters, the upper row represents the first set of measurements, the lower row the second set of measurements. (PDF) [file pone.0200695.s006.pdf]

S4 Table. Descriptive and reproducibility results of the express saccadic task

Parameter	Mean	SD	Range	ICC (95% CI)	CR	CV (%)
Peak velocity (deg/s)	327<br>332	49<br>48	246 – 434<br>255 – 419	0.88 (0.72 – 0.96)	33	3.6
Peak acceleration (deg/s^2)	31673<br>32137	5162<br>4815	22706 – 42095<br>23859 – 42576	0.82 (0.58 – 0.93)	4113	4.6
Latency (ms)	138<br>132	24<br>16	102 – 204<br>104 – 154	0.76 (0.44 – 0.91)	17	4.3
Gain	0.95<br>0.96	0.06<br>0.06	0.82 – 1.08<br>0.77 – 1.05	0.76 (0.46 – 0.90)	6.5	2.5
