# Supplementary material for: A standardized protocol for quantification of saccadic eye movements: DEMoNS
Source: PLoS One. 2018 Jul 16;13(7):e0200695. doi: 10.1371/journal.pone.0200695 (PMC6047815; doi:10.1371/journal.pone.0200695)
Supplement: S5 Table — FS: first saccade, SS: second saccade, DS: double-step saccade, deg: degrees, s: seconds, ms: milliseconds, SD: standard deviation, ICC: intra-class correlation coefficient, CI: confidence interval, CV: coefficient of variation, CR: coefficient of repeatability. For every parameters, the upper row represents the first set of measurements, the lower row the second set of measurements. (PDF) [file pone.0200695.s007.pdf]

S5 Table. Descriptive and reproducibility results of the double-step saccadic task

Parameter	Mean	SD	Range	ICC (95% CI)	CR	CV (%)
Peak velocity FS (deg/s)	300	52	222 – 403	0.87 (0.69 – 0.95)	41	4.8
	309	53	233 – 416			
Peak acceleration FS (deg)	30816	5311	23811 – 41875	0.85 (0.66 – 0.94)	4727	5.4
	31610	5583	24194 – 43626			
Latency FS (ms)	279	73	179 – 439	0.88 (0.63 – 0.96)	42	5.6
	263	55	183 – 374			
Latency correct FS (ms)	269	73	159 – 412	0.91 (0.70 – 0.97)	46	6.4
	253	62	153 – 376			
Amplitude FS (deg)	6.8	1.1	5.1 – 8.9	0.85 (0.64 -0.94)	1.0	5.2
	6.9	0.9	5.5 – 8.9			
Direction difference FS (deg)	16.2	7.7	8.4 – 38.6	0.55 (0.16 – 0.80)	9.8	24.1
	13.6	5.2	6.6 – 26.6			
Peak velocity SS (deg)	279	54	187 – 414	0.86 (0.68 – 0.94)	50	6.6
	285	59	205 – 409			
Peak acceleration SS (deg/s^2)	28334	5544	19471 – 42084	0.86 (0.67 – 0.94)	4983	6.6
	28734	6259	18381 – 43125			
Intersaccadic interval (ms)	431	115	203 – 748	0.75 (0.45 – 0.90)	134	11.7
	391	121	150 – 583			
Intersaccadic interval correct SS (ms)	404	115	203 – 586	0.73 (0.43 – 0.89)	128	11.9
	386	114	150 – 568			
Gain SS	0.88	0.08	0.69 – 1.04	0.65 (0.04 – 0.87)	0.12	4.6
	0.93	0.07	0.75 – 1.02			
Direction difference SS (deg)	21.3	7.7	10.0 – 37.3	0.59 (0.21 – 0.82)	9.4	16.2
	19.0	5.9	9.0 – 30.1			
Gain FEP	0.97	0.09	0.74 – 1.14	0.55 (0.17 – 0.80)	0.12	4.6
	1.00	0.09	0.70 – 1.13			
X error FEP (deg)	0.24	0.74	-1.95 – 1.48	0.81 (0.57 – 0.92)	0.68	N/A
	0.28	0.63	-1.01 – 1.81			
Y error FEP (deg)	-0.38	0.59	-1.88 – 0.57	0.57 (0.18 – 0.81)	0.74	N/A
	-0.19	0.49	-1.52 – 0.60			

XY absolute error FEP (deg)	1.47	0.62	0.63 – 2.70	0.77 (0.32 – 0.92)	0.63	16.1
	1.22	0.51	0.46 – 2.13			
Proportion correct DS	0.62	0.24	0.13 – 0.93	0.63 (0.24 – 0.84)	0.29	22.0
	0.66	0.16	0.42 – 0.92			
Proportion acceptable DS	0.64	0.25	0.18 – 0.97	0.65 (0.29 – 0.85)	0.27	26.7
	0.72	0.16	0.45 – 0.97			
Proportion contraversive DS	0.03	0.05	0.00 – 0.24	0.73 (0.42 – 0.89)	0.04	N/A
	0.03	0.03	0.00 – 0.13			
Proportion late DS	0.05	0.08	0.00 – 0.25	0.63 (0.24 – 0.84)	0.06	N/A
	0.02	0.04	0.00 – 0.15			
Proportion FS to 2nd target	0.09	0.11	0.02 – 0.48	0.71 (0.38 – 0.88)	0.10	N/A
	0.07	0.07	0.00 – 0.32			
