# Supplementary material for: A standardized protocol for quantification of saccadic eye movements: DEMoNS
Source: PLoS One. 2018 Jul 16;13(7):e0200695. doi: 10.1371/journal.pone.0200695 (PMC6047815; doi:10.1371/journal.pone.0200695)
Supplement: S6 Table — VDI: versional dysconjugacy index, deg: degrees, s: seconds, ms: milliseconds, SD: standard deviation, ICC: intra-class correlation coefficient, CI: confidence interval, CV: coefficient of variation, CR: coefficient of repeatability. For every parameters, the upper row represents the first set of measurements, the lower row the second set of measurements. (PDF) [file pone.0200695.s008.pdf]

S6 Table. Descriptive and reproducibility results of the repeated pro-saccadic task

Parameter	Mean	SD	Range	ICC (95% CI)	CR	CV (%)
Peak velocity (deg/s)	321	46	236 – 420	0.81 (0.58 – 0.92)	38	4.3
	327	42	261 – 412			
Peak acceleration (deg/s^2)	30866	4779	22330 – 41163	0.78 (0.52 – 0.91)	4064	4.7
	31463	4639	23386 – 41883			
Latency (ms)	180	23	151 – 233	0.88 (0.71 – 0.95)	15	3.0
	176	18	149 – 219			
Gain	0.96	0.05	0.84 – 1.08	0.65 (0.29 – 0.85)	0.07	2.5
	0.97	0.04	0.86 – 1.05			
Peak velocity / amplitude	41.2	5.0	32.7 – 49.3	0.81 (0.58 – 0.92)	4.0	3.5
	41.8	5.1	33.7 – 51.2			
VDI peak velocity	1.08	0.08	0.92 – 1.24	0.94 (0.86 – 0.98)	0.05	1.6
	1.09	0.09	0.92 – 1.26			
VDI peak velocity left	1.09	0.10	0.93 – 1.26	0.94 (0.85 – 0.98)	0.06	2.0
	1.10	0.10	0.96 – 1.34			
VDI peak velocity right	1.08	0.09	0.91 – 1.21	0.81 (0.57 – 0.92)	0.08	2.8
	1.08	0.09	0.86 – 1.22			
VDI peak acceleration	1.10	0.12	0.90 – 1.31	0.90 (0.75 – 0.96)	0.08	2.7
	1.11	0.12	0.92 – 1.34			
VDI peak acceleration left	1.12	0.16	0.86 – 1.41	0.93 (0.83 – 0.97)	0.10	3.2
	1.12	0.16	0.90 – 1.40			
VDI peak acceleration right	1.09	0.11	0.93 – 1.30	0.70 (0.39 – 0.87)	0.13	4.2
	1.12	0.10	0.95 – 1.28			
VDI FPG	1.04	0.05	0.96 – 1.16	0.84 (0.64 – 0.94)	0.04	1.2
	1.05	0.05	0.96 – 1.16			
VDI FPG left	1.04	0.05	0.96 – 1.18	0.81 (0.57 – 0.92)	0.05	1.7
	1.05	0.06	0.97 – 1.23			
VDI FPG right	1.04	0.05	0.96 – 1.14	0.80 (0.55 – 0.92)	0.04	1.3
	1.05	0.05	0.95 – 1.14			
VDI AUC	1.10	0.07	0.97 – 1.24	0.94 (0.86 – 0.98)	0.04	1.2
	1.10	0.07	0.97 – 1.26			

VDI AUC left	1.11	0.09	0.98 – 1.29	0.92 (0.81 – 0.97)	0.06	1.9
	1.11	0.08	1.02 – 1.27			
VDI AUC right	1.08	0.07	0.95 – 1.19	0.80 (0.55 – 0.92)	0.08	2.6
	1.08	0.08	0.88 – 1.26			
VDI peak velocity / amplitude	1.05	0.05	0.96 – 1.16	0.88 (0.69 – 0.95)	0.04	1.4
	1.07	0.06	0.97 – 1.19			
VDI peak velocity / amplitude left	1.06	0.06	0.94 – 1.17	0.75 (0.47 – 0.90)	0.06	2.0
	1.07	0.07	0.98 – 1.23			
VDI peak velocity / amplitude right	1.05	0.07	0.95 – 1.17	0.89 (0.75 – 0.96)	0.05	1.7
	1.06	0.07	0.96 – 1.21			
