# Supplementary material for: A standardized protocol for quantification of saccadic eye movements: DEMoNS
Source: PLoS One. 2018 Jul 16;13(7):e0200695. doi: 10.1371/journal.pone.0200695 (PMC6047815; doi:10.1371/journal.pone.0200695)
Supplement: S7 Table — BCEA: bivariate contour ellipse area, IQR: interquartile range, SE: standard error of the estimate, SWJ: square wave jerk, VDI: versional dysconjugacy index, FPG: first-pass gain, AUC: area under the curve, AS: anti-saccades, PS: pro-saccades, FEP: final eye position, FS: first saccade, SS: second saccade, DS: double-step saccade, deg: degrees, s: seconds, ms: milliseconds, nr: number, SD: standard deviation. (PDF) [file pone.0200695.s009.pdf]

S7 Table. Descriptive results of all tasks of center 2.

Parameter	Mean	SD	Range
Fixation task			
SD X gaze (deg)	0.21	0.09	0.12 – 0.37
SD Y gaze (deg)	0.25	0.09	0.16 – 0.40
BCEA gaze (deg^2)	0.28	0.18	0.13 – 0.63
SD X vergence (deg)	0.21	0.07	0.14 – 0.35
SD Y vergence (deg)	0.26	0.07	0.14 – 0.40
BCEA vergence (deg^2)	0.33	0.19	0.11 – 0.71
Mean velocity X (deg/s)	0.01	0.07	-0.13 – 0.16
SD velocity X (deg/s)	1.96	0.37	1.54 – 2.72
Mean velocity Y (deg/s)	0.04	0.07	-0.05 – 0.17
SD velocity Y (deg/s)	2.62	0.57	1.90 – 3.62
Median velocity total (deg/s)	2.19	0.41	1.57 – 2.74
IQR velocity total (deg/s)	2.16	0.44	1.60 – 2.95
Linear fit coefficient X gaze (deg/s)	0.01	0.07	-0.11 – 0.15
SE linear fit X gaze (deg)	0.16	0.06	0.11 – 0.29
Linear fit coefficient Y gaze (deg/s)	0.04	0.07	-0.05 – 0.16
SE linear fit Y gaze (deg)	0.21	0.07	0.15 – 0.35
Linear fit coefficient X vergence (deg/s)	0.03	0.08	-0.05 – 0.25
SE linear fit X vergence (deg)	0.21	0.08	0.11 – 0.38
Linear fit coefficient Y vergence (deg/s)	0.02	0.07	-0.14 – 0.16
SE linear fit Y vergence (deg)	0.27	0.09	0.13 – 0.43
SWJ >4 deg (nr/s)	0.0	0.0	0.0 – 0.0
SWJ <4 deg (nr/s)	0.37	0.33	0.05 – 1.09
Mean amplitude SWJ (deg)	0.55	0.31	0.27 – 1.29
Saccades >2 deg (nr/s)	0.02	0.02	0.00 – 0.07
Saccades <2 deg (nr/s)	0.63	0.18	0.20 – 0.80
Mean amplitude saccades (deg)	0.54	0.26	0.26 – 1.16

Mean amplitude SWJ + saccades (deg)	0.53	0.20	0.33 – 1.07
Mean duration intra-SWJ interval (ms)	159	49	97 – 259
Pro-saccades task			
Peak velocity (deg/s)	336	37	279 – 394
Peak velocity 15 deg (deg/s)	373	43	302 – 442
Peak velocity 8 deg (deg/s)	297	35	252 – 348
Peak acceleration (deg/s^2)	20857	3742	15957 – 26782
Peak acceleration 15 deg (deg/s^2)	22391	4178	17179 – 29551
Peak acceleration 8 deg (deg/s^2)	19265	3414	13584 – 24298
Latency (ms)	172	17	134 – 202
Latency 15 deg (ms)	180	23	138 – 222
Latency 8 deg (ms)	162	14	131 – 182
Gain	0.94	0.04	0.84 – 0.98
Gain 15 deg	0.92	0.04	0.82 – 0.96
Gain 8 deg	0.97	0.05	0.86 – 1.02
VDI peak velocity	1.08	0.07	0.95 – 1.22
VDI peak velocity 15 deg	1.08	0.07	0.96 – 1.21
VDI peak velocity 8 deg	1.08	0.08	0.94 – 1.26
VDI peak velocity left	1.11	0.14	0.92 – 1.31
VDI peak velocity right	1.05	0.05	0.96 – 1.13
VDI peak acceleration	1.11	0.10	0.95 – 1.30
VDI peak acceleration 15 deg	1.12	0.10	0.96 – 1.29
VDI peak acceleration 8 deg	1.10	0.10	0.95 – 1.31
VDI peak acceleration left	1.15	0.14	0.92 – 1.41
VDI peak acceleration right	1.07	0.07	0.97 – 1.18
VDI FPG	1.03	0.02	0.99 – 1.07
VDI FPG 15 deg	1.02	0.02	0.99 – 1.05
VDI FPG 8 deg	1.03	0.03	0.99 – 1.09
VDI FPG left	1.02	0.02	0.99 – 1.05
VDI FPG right	1.03	0.02	0.99 – 1.08
VDI AUC	1.08	0.05	0.99 – 1.17
VDI AUC 15 deg	1.08	0.05	0.99 – 1.14

VDI AUC 8 deg	0.09	0.05	0.98 – 1.19
VDI AUC left	1.10	0.07	0.97 – 1.23
VDI AUC right	1.06	0.04	1.00 – 1.11
Anti-saccade task			
Peak velocity CS (deg/s)	274	71	175 – 282
Peak acceleration CS (deg/s^2)	18338	5064	11697 – 25508
Latency (ms)	197	35	144 – 258
Latency AS (ms)	212	33	159 – 258
Latency PS (ms)	172	77	97 – 338
Gain AS	0.95	0.25	0.47 – 1.33
X error AS (deg)	-0.55	2.15	-4.76 – 3.01
Proportion errors	0.22	0.12	0.08 – 0.50
Latency correction PS (ms)	252	140	95 – 569
Gain FEP	1.00	0.10	0.82 – 1.20
X error FEP	-0.07	0.81	-1.58 – 1.77
Express saccade task			
Peak velocity (deg/s)	282	31	241 – 332
Peak acceleration (deg/s^2)	18405	2886	14163 – 21884
Latency (ms)	120	22	85 – 160
Gain	0.93	0.06	0.82 – 1.06
Double-step saccade task			
Peak velocity FS (deg/s)	250	53	183 – 331
Peak acceleration FS (deg)	17207	4210	11047 – 23410
Latency FS (ms)	253	44	204 – 322
Latency correct FS (ms)	244	47	193 – 329
Amplitude FS (deg)	7.17	0.89	5.36 – 8.53
Direction difference FS (deg)	16.77	5.33	7.02 – 26.71
Peak velocity SS (deg)	230	34	175 – 284
Peak acceleration SS (deg/s^2)	15375	2994	10487 – 21182
Intersaccadic interval (ms)	328	121	176 – 553
Intersaccadic interval correct SS (ms)	331	117	190 – 552
Gain SS	0.95	0.13	0.81 – 1.23
Direction difference SS (deg)	19.20	6.03	9.76 – 29.53

Gain FEP	1.03	0.12	0.89 – 1.30
X error FEP (deg)	0.28	0.64	-0.72 – 1.72
Y error FEP (deg)	0.02	0.73	-0.76 – 1.50
XY absolute error FEP (deg)	1.42	0.45	0.95 – 2.44
Proportion correct DS	0.51	0.13	0.25 – 0.73
Proportion (almost) correct DS	0.67	0.15	0.41 – 0.90
Proportion contraversive DS	0.02	0.03	0.00 – 0.09
Proportion late DS	0.01	0.02	0.00 – 0.07
Proportion FS to 2nd target	0.06	0.06	0.02 – 0.25
Repeated pro-saccades			
Peak velocity (deg/s)	279	32	232 – 330
Peak acceleration (deg/s^2)	17978	2920	13702 – 21471
Latency (ms)	162	19	131 – 206
Gain	0.94	0.04	0.87 – 1.02
VDI peak velocity	1.08	0.08	0.96 – 1.25
VDI peak velocity left	1.11	0.09	0.94 – 1.29
VDI peak velocity right	1.06	0.07	0.96 – 1.21
VDI peak acceleration	1.10	0.10	0.93 – 1.30
VDI peak acceleration left	1.12	0.12	0.87 – 1.31
VDI peak acceleration right	1.07	0.10	0.92 – 1.29
VDI FPG	1.03	0.02	1.00 – 1.09
VDI FPG left	1.03	0.02	0.99 – 1.06
VDI FPG right	1.04	0.03	1.00 – 1.11
VDI AUC	1.08	0.06	0.99 – 1.19
VDI AUC left	1.11	0.07	0.96 – 1.23
VDI AUC right	1.05	0.06	0.97 – 1.16
