# Supplementary figures and images for: A standardized protocol for quantification of saccadic eye movements: DEMoNS
Source: PLoS One. 2018 Jul 16;13(7):e0200695. doi: 10.1371/journal.pone.0200695 (PMC6047815; doi:10.1371/journal.pone.0200695)

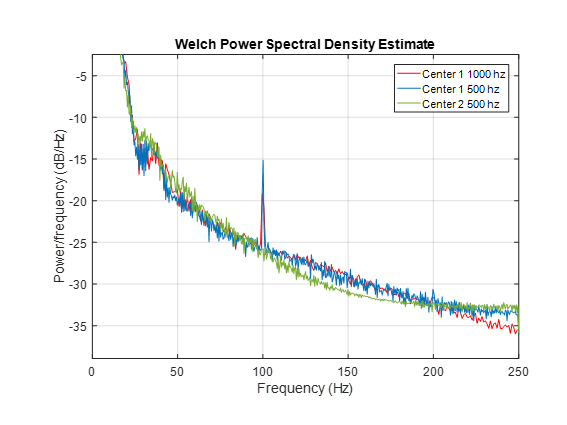

Supplement: S1 Fig — Data of one subject measured in both centers. The red line represents the data from center one at the original sampling frequency of 1000 Hz, the blue line the same data downsampled to 500 Hz and the green line the data of center two sampled at 500 Hz. (TIF) [file pone.0200695.s010.tif]

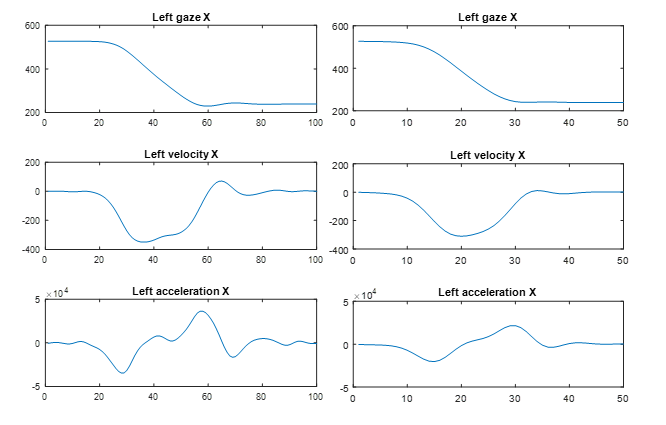

Supplement: S2 Fig — Signal (filtered data of center one) of a leftward 8 degrees saccade, the same saccade is shown at 1000 Hz (left) and downsampled to 500 Hz (right). (TIF) [file pone.0200695.s011.tif]
